# Lymphocyte Antigen 6G Mediates Vagotomy‐Associated Reduction in Body Weight

**DOI:** 10.1096/fj.202600151RR

**Published:** 2026-04-04

**Authors:** Ting Liu, April S. Caravaca, Min Cai, Wanmin Dai, Jérômine Jeanne Vacquié, Qi Guo, Peter Holicek, Maxime Villet, Patricia Teixeira Mendes, Yuyang Zhang, Rubén R. Simón, Shengduo Pei, Fredrik Wermeling, Stephen G. Malin, Mikael C. I. Karlsson, Vladimir S. Shavva, Carolina E. Hagberg, Laura Tarnawski, Peder S. Olofsson

**Affiliations:** ^1^ Laboratory of Immunobiology, Center for Bioelectronic Medicine, Division of Cardiovascular Medicine, Department of Medicine, Solna, Center for Molecular Medicine Karolinska Institutet Stockholm Sweden; ^2^ Division of Cardiovascular Medicine, Department of Medicine, Solna, Center for Molecular Medicine Karolinska Institutet Stockholm Sweden; ^3^ Department of Microbiology, Tumor and Cell Biology Karolinska Institutet Solna Sweden; ^4^ Division of Rheumatology, Department of Medicine, Solna Karolinska Institutet, and Karolinska University Hospital Solna, and Center for Molecular Medicine, Karolinska University Hospital Stockholm Sweden

**Keywords:** Ly6G, neutrophils, vagotomy, weight loss

## Abstract

Dysregulation of adipocyte function is an important component of metabolic and cardiovascular diseases. It is increasingly evident that interorgan neuroimmune crosstalk in adipose tissue maintains adipose tissue functionality, yet the molecular mechanisms are incompletely understood. The vagus nerve regulates numerous physiological functions, including inflammation and weight control, even in tissues lacking direct cholinergic innervation. In this study, we examined whether vagal signaling is involved in maintaining baseline homeostasis in epididymal white adipose tissue (eWAT) by investigating how disrupting vagus signaling affects adipose tissue physiology. As expected, vagotomized male animals had lower body weight and epididymal white adipose tissue mass as compared with controls. Vagotomized animals showed increased Ly6G^+^ cell infiltration in eWAT. Interestingly, vagotomy‐associated weight loss was significantly attenuated in neutrophil‐deficient Ly6G^cre^Mcl1^fl/fl^ mice and in mice treated with repeated injections of anti‐Ly6G antibodies. Together, these observations indicate that there is a Ly6G‐mediated vagotomy‐associated reduction in weight and reveal that Ly6G^+^ cells participate in the regulation of eWAT energy homeostasis.

## Graphical abstract

Vagotomy was associated with lower body weight and epididymal white adipose tissue (eWAT) mass, as well as increased Ly6G^+^ cell infiltration in eWAT. This vagotomy‐associated weight loss was significantly attenuated in neutrophil‐deficient mice, suggesting Ly6G^+ ^cells participate in the regulation of eWAT energy homeostasis.

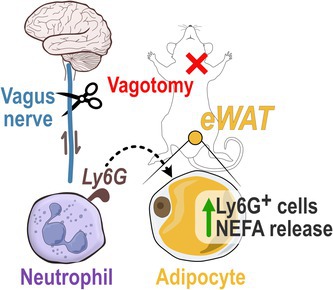

## 1 Introduction

Adipose tissue is a key metabolic organ central for homeostasis and health. Dysregulation of adipocyte function is an important component of several metabolic diseases and cardiovascular diseases [1, 2]. It is evident that interorgan crosstalk in adipose tissue is important for its function, homeostasis, and health [3, 4, 5]. Signals in the largely cholinergic vagus nerve are known to regulate adipose tissue function, and weight loss after vagotomy has been consistently observed across multiple species [6, 7, 8, 9, 10, 11]. However, the mechanism remains unclear since vagal innervation of epididymal white adipose tissue (eWAT) is very sparse or absent [12]. Intriguingly, there is evidence that vagal regulation of physiological functions involves intermediary immune cells that relay neural signals in organs that, similar to eWAT, largely lack direct cholinergic innervation [13, 14, 15, 16]. Despite these insights, little is known about the early temporal dynamics of cervical vagus‐associated regulation of noninflamed eWAT physiology. Several subtypes of neurons have been reported to innervate white adipose tissue, the majority being adrenergic. Norepinephrine released from adrenergic neurons in adipose tissue interacts with local macrophages and promotes lipolysis [3, 4, 17, 18]. However, blocking monocyte recruitment to adipose tissue did not affect body weight, suggesting that monocytes/macrophages are not the sole immune cells involved in neural regulation of adipocyte function [19]. Neutrophils, key mediators of the innate immune response, have historically received limited attention in the context of the adipose tissue microenvironment. However, evidence from animal models of obesity suggests they may play a pivotal role in initiating adipose inflammation and insulin resistance [20, 21, 22]. Murine neutrophils can be identified by their expression of Ly6G, a member of the Ly6/uPAR family of GPI‐anchored surface proteins. While the natural ligand for Ly6G remains unknown, its role in neutrophil migration has been well documented [23, 24]. Here, we investigated the early eWAT response to unilateral vagotomy in healthy mice and unexpectedly uncovered a role for Ly6G in the regulation of eWAT homeostasis.

## 2 Materials and Methods

### 2.1 Ethics Statement

This study and all experimental protocols were approved by the regional Stockholm Animal Research Ethics Committee (Stockholm, Sweden).

### 2.2 Animals

Male (age 10–17 weeks) C57BL/6 (wild‐type, Charles River Laboratories) and *Ly6g* ^cre^ (C57BL/6 background, B6. Ly6gtm2621(cre)Arte) *Mcl1* ^flox/flox^ (C57BL/6 background, B6.129‐Mcl1tm3Sjk/J) mice (a generous gift from Oliver Söhnlein lab (University of Münster, Germany)) were housed under a 12‐h light/dark cycle with *ad libitum* access to standard chow and water. Male mice were chosen as female mice do not have epididymal adipose tissue (eWAT).

### 2.3 Food Consumption Measurement

Weight‐ and age‐matched male mice were divided into 4 cages based on treatment group. The food in the cage was weighed (Sartorius, #BA2105) daily, and the consumption of food was calculated as grams per day per cage.

### 2.4 Vagotomy Surgery

Cervical vagus nerve isolation and vagotomy was preformed as described previously [25]. In brief, following the induction of anesthesia with isoflurane at 3% and a 1:1 mixture of oxygen and air, the isoflurane level was reduced to 1.5% for maintenance. The fur of the neck of the mouse was shaved and cleaned using 70% ethanol. Then, following a ventral midline cervical incision, the subcutaneous tissue and salivary glands were exposed and retracted. The left cervical vagus nerve was isolated away the vasculature and immobilized with a suture. Forceps were used to lift and hold the vagus nerve, and a 2–3 mm nerve segment was cut away from the nerve. Salivary glands and tissues were then repositioned, and the skin was sutured. Sham mice had their cervical vagus nerve exposed, and the incision was closed.

### 2.5 Non‐Esterified Fatty Acids (NEFA) and Glycerol Quantification

Whole blood from the inferior vena cava was collected in 1.5 mL tubes, incubated at room temperature (RT) for 30 min, and centrifuged at 600 g for 7 min at RT. Supernatants were collected, transferred to new 1.5 mL tubes and centrifuged at 10600 g for 1 min at RT. Serum was collected and stored at −80°C. NEFA levels were quantified by using the NEFA kit (Fujifilm Wako Chemicals Europe, #434–91 795, #436–91 995, #270–77 000) (*n* = 1 experiment). Glycerol was quantified using the Free Glycerol Assay Kit (Abcam, #ab65337) (*n* = 2 experiments).

### 2.6 Ex Vivo NEFA and CCL2 Release From eWAT

eWAT was collected from male C57BL/6 mice, weighed (Sartorius, #BA2105), and dissected into ~20 mg fragments using a sterile scalpel. Tissue pieces were rinsed with 2 mL phosphate‐buffered saline (PBS; Gibco, #10010023) and transferred into Eppendorf tubes containing 500 μL Dulbecco's Modified Eagle Medium (DMEM; Gibco, #31966–021) supplemented with 2% fatty acid–free bovine serum albumin (BSA; Sigma‐Aldrich, #A4503‐100 g). Samples were incubated at 37°C in a pre‐warmed shaker at 160 rpm for 3 h and the supernatants were collected for the following analysis: (1) NEFA levels were quantified using the NEFA kit (Wako Chemicals). In vitro release rate was calculated as mg NEFAs per mg eWAT tissue per hour. (2) CCL2 levels were quantified using a mouse CCL2 ELISA kit (R&D Systems, #DY497‐05), according to the manufacturer's instructions (*n* = 1 experiment). CCL2 release was normalized to tissue weight and expressed as ng/mL per mg of eWAT.

### 2.7 Bulk RNA Sequencing

C57BL/6 mice were subjected to sham surgery or vagotomy. After 7 days, bone marrow neutrophils were isolated using magnetic beads (Miltenyi Biotec, #130‐097‐658). Total RNA was extracted from the neutrophils using Quick‐RNA Microprep Kit (Zymo, #R1050). Pair‐end mRNA sequencing of poly‐A‐enriched RNA (30 million reads) was performed at Novogene. Alignment (mouse transcriptome release M31, GRCm39) and quantification were performed using Salmon [26]. RNA‐sequencing analysis was performed using DESeq2 [27]. Pheatmap was used to generate heatmaps. ClusterProfiler was used to perform pathway analysis [28] (*n* = 2 experiments). Code will be deposited at https://github.com/ImmunoBioLab/ upon publication.

### 2.8 Isolation of Stromal Vascular Cells From eWAT

eWAT was isolated, weighed (Sartorius, #BA2105), and minced manually using scissors. A single cell suspension was obtained by digestion (2% BSA, 1.4 mg/mL collagenase (Sigma, #C6885)) in HBSS (Gibco, #14175–053) at 37°C with agitation (160 rpm/min) for 12 min. Digestion was quenched with 10 mL DMEM (Gibco, #21969–035) with 10% FBS (ThermoFisher, #10270106). Samples were filtered (100 μm pore size filter) and centrifuged at 600 g, RT, 5 min. Cell pellets were resuspended in 500 μL red blood cell lysis buffer (QIAgen, #79217) for 5 min at RT and diluted with 5 mL DMEM with 10% FBS. Cells were centrifuged at 600 g, RT, 5 min and washed with 3 mL of PBS (without Ca^2+^ or Mg^2+^). The cells were resuspended in PBS (−/−).

### 2.9 Flow Cytometry Analysis

SVCs were incubated with anti‐mouse CD16/CD32 mouse FC receptor block (BD Biosciences, #553142) for 15 min, and stained with antibodies (Table S1) and viability dye Zombie Aqua Fixable Viability Kit for 30 min at RT. For the detection of intracellular Ly6G, cells were fixed and permeabilized using Foxp3/Transcription Factor Staining buffer set (eBioscience, #00–5521‐00) following staining with extracellular antibodies. Flow cytometry was performed using Cytek Northern Lights (Cytek, #NL‐3000).

### 2.10 Immunofluorescence

eWAT was collected from male mice and fixed using 4% formaldehyde (VWR, #9713.1000) for 24 h. Samples were dehydrated in 70% ethanol (VWR, #83801.290) using the microwave hybrid tissue processor (Milestone, LOGOS). Next, samples were embedded in paraffin, sectioned (5 μm), and rehydrated. Antigen retrieval was performed using the antigen retrieval solution (DIVA Decloaker, pH 6, #DV2004, BiocareMedical; 10×, diluted in MilliQ). Sections were blocked in 5% horse serum in 0.05% TBST for 30 min at RT. eWAT paraffin section were stained with rabbit anti‐mouse anti‐perilipin 1 (Cell signaling technology, #9349 T) and rat anti‐mouse anti‐Ly6G antibody (BD Biosciences, #551459), washed with 0.05% TBST, and incubated with secondary antibodies (horse anti‐rabbit IgG Antibody (H + L), DyLight 488 (Vector Laboratories, #DI‐1088), and Goat Anti‐Rat IgG Antibody (H + L) DyLight 594 (Abcam, #ab98422)) or horse anti‐rabbit IgG antibody (H+L), DyLight 594 (Vector Laboratories, #DI‐1094). Nuclei were stained with DAPI (4′,6‐diamidino‐2‐phenylindole), and samples were mounted with Dako mounting media (Agilent). Images were acquired with a Leica Stellaris 5 system microscope and further processed with ImageJ FIJI software.

### 2.11 Whole Tissue Staining of eWAT With BODIPY FL C12

eWAT was isolated from male mice and stained with BODIPY FL C12 (Invitrogen, #D3822, 1:2000) and DAPI (Thermo scientific, #D1306, 1:5000) in 0.1% TBST for 60 min at RT. Samples were washed with PBS three times and transferred to the chamber (Lab‐Tek Chamber Slide System, Thermo Scientific, #177437). Images were acquired using a Nikon Eclipse T12 confocal microscope. Cell size was quantified using an automated macro for ImageJ FIJI software. *n* = 2 experiments.

### 2.12 Hematoxylin/Eosin Staining

eWAT was isolated from male mice and fixed in 4% PFA for 48 h. Samples were dehydrated in 75% ethanol and embedded in paraffin. Paraffin sections (5 μm) were rehydrated and stained with hematoxylin and eosin before mounting and microscopic imaging. *n* = 1 experiment. Stained sections were examined by ZEISS AxioVision microscope at 20* magnification. Adipocyte size and count was measured using the Adiposoft [29] plugin in ImageJ FIJI software, with parameters set to 20–120 μm. Nuclei count was measured in ImageJ FIJI by threshold and particles counting, then normalized by the following equation: Non‐adipocyte nuclei % = ((Total nuclei count—Adipocyte count)/Total nuclei count) × 100%.

### 2.13 Anti‐Ly6G Antibody Treatment

To ligate Ly6G surface epitopes, male C57BL/6 mice were intraperitoneally injected with InVivo Mab anti‐mouse Ly6G (100 ug/100 uL) (Bioxcell, #BE0075) or for control InVivoMAb rat IgG2a (anti‐trinitrophenol) isotype (100 ug/100 uL) (Bioxcell, #BE0089) antibodies 2 days before the sham or VX surgery. To achieve transient ligation of Ly6G, only this injection was administered (*n* = 1 experiment). For sustained ligation of Ly6G, injections were repeated at days 3 and 5 after surgery (*n* = 2 experiments). Mice were euthanized and tissues were collected 7 days after the sham or VX surgery.

### 2.14 Statistical Analysis and Study Design

Methods used for statistical analysis are described in the figure legends. The statistical calculations were performed using GraphPad Prism 10.4.1 (GraphPad Software). Outliers were identified using Grubbs's test. Outliers are shown as red dots. *p* ≤ 0.05 was considered statistically significant. Animals were assigned randomly to various experimental groups by the researcher, following a pre‐determined template decided upon before interaction with the mice, whenever possible. Researchers were blind to group assignments for data collection and analysis, whenever possible. In place of a power analysis, a resource allocation equation was used.

## 3 Results

### 3.1 Vagotomy Reduced eWAT Weight and Promoted NEFA Release

**FIGURE 1:**
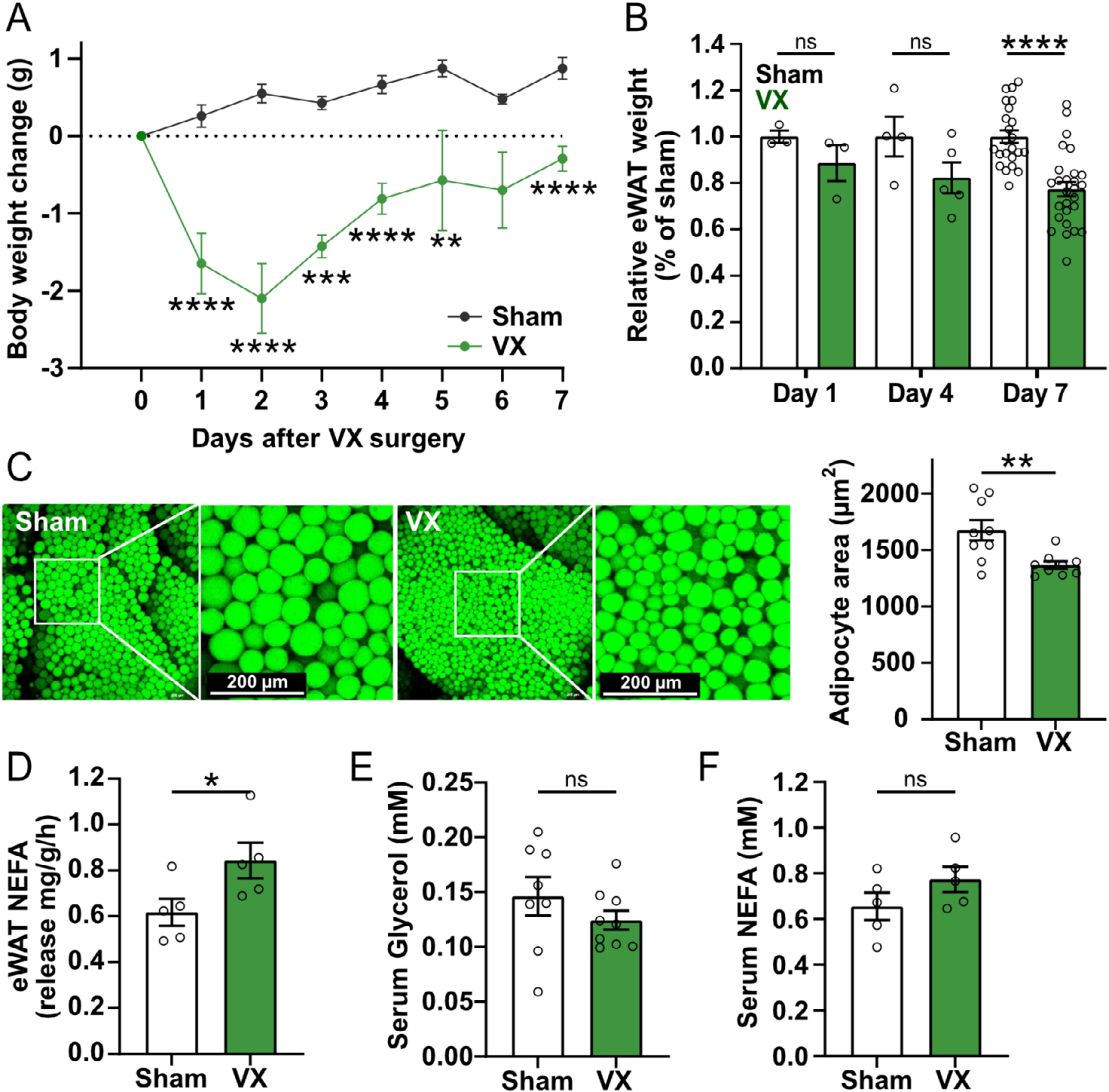
Vagotomy reduced eWAT weight. Wild‐type mice were subjected to either left cervical vagotomy (VX) or sham surgery. (A) The mice were weighed before (day 0, *n* = 6–7) and at 1 (*n* = 6–7), 2 (*n* = 4), 3 (*n* = 4), 4 (*n* = 8), 5 (*n* = 4), 6 (*n* = 4), and 7 (*n* = 18–19) days following VX or sham surgery. The graph shows the change in body weight of the mice from day 0 (before initiation of surgery) of each experimental group in g ± SEM (two‐way ANOVA, Šídák's multiple comparisons test). (B) eWAT weight was recorded at 1 (*n* = 3), 4 (*n* = 4 sham, *n* = 5 VX), and 7 (*n* = 23, *n* = 27 VX) days following VX or sham surgery. The bars depict the ratio of eWAT weight to sham eWAT weight in % ± SEM (One‐way ANOVA, Uncorrected Fisher's LSD). (C) eWAT was collected at 7 days after sham surgery or VX and stained using BODIPY FL C12. Left panel shows representative images of the whole eWAT staining. Adipocyte cell size was quantified using image J. In the right panel, each dot represents the average adipocyte cell size in sham (*n* = 9) and VX (*n* = 9) eWAT shown as μm2 ± SEM (unpaired Student's *t* test). (D) eWAT was collected at 7 days after sham (*n* = 5) or VX (*n* = 5) surgery and NEFA release from eWAT was quantified. The bar depicts the NEFAs released from eWAT normalized by eWAT weight per hour in mg/g/h ± SEM (unpaired Student's *t* test). (E‐F) Serum was collected 7 days after sham or VX surgery. (E) Glycerol (*n* = 8 sham, *n* = 9 VX) and (F) NEFAs (*n* = 5) were quantified. The bars depict mM ± SEM (unpaired Student's *t* test). eWAT, epididymal white adipose tissue; VX, Vagotomy; ns = not significant, a.u.—arbitrary units; *p < 0.05, **p < 0.01, ****p < 0.0001. NEFA, non‐esterified fatty acid.

Vagotomy (VX) is known to promote weight loss [6, 7, 8, 9, 10, 11] as well as a reduction in white adipose tissue mass in rats and humans [30, 31]. To study the early temporal dynamics of vagus nerve‐mediated reduction in epididymal white fat tissue (eWAT) weight under conditions without induced inflammation, C57BL/6 mice were subjected to left cervical VX or sham surgery. Mice subjected to VX had a significantly lower body weight at 1, 2, 3, 4, and 7 days as compared to before surgery (day 0) (Figure 1A), while the sham‐treated animals significantly increased in weight by 7 days post‐surgery (Figure S1A–C). Furthermore, we observed that eWAT weight was significantly lower at 7 days following VX compared with sham‐treated mice (Figure 1B).

BODIPY FL C12 staining of eWAT showed that adipocyte area was significantly lower following VX compared with sham treatment (Figure 1C, S1D,E). Accordingly, we next investigated whether VX promoted adipocyte lipolysis. eWAT isolated from mice following VX released more non‐esterified fatty acids (NEFAs) than eWAT isolated from sham‐treated animals (Figure 1D). No significant difference in glycerol or NEFAs in the blood was found between VX‐ and sham‐treated animals suggesting this could be a local effect (Figure 1E). Taken together, vagotomy reduced epididymal fat mass through enhanced local lipolysis as evidenced by decreased adipocyte size and increased ex vivo NEFA release.

### 3.2 Vagotomy Promoted Neutrophil Infiltration Into eWAT

It is well established that the vagus nerve regulates inflammation [13, 14, 32] and immune cell activation is a well‐known driver of adipose tissue lipolysis [21, 33]. Accordingly, we compared the immune cell repertoire in blood, bone marrow, and spleen between VX‐ and sham‐treated mice and observed no significant differences in proportions of CD11b^+^Ly6G^+^, CD11b^+^Ly6G^−^F4/80^+^, CD11b^−^Ly6G^−^CD19^+^CD3^−^, and CD11b^−^Ly6G^−^CD19^−^CD3^+^ 7 days after surgery (Figure S2A).

**FIGURE 2:**
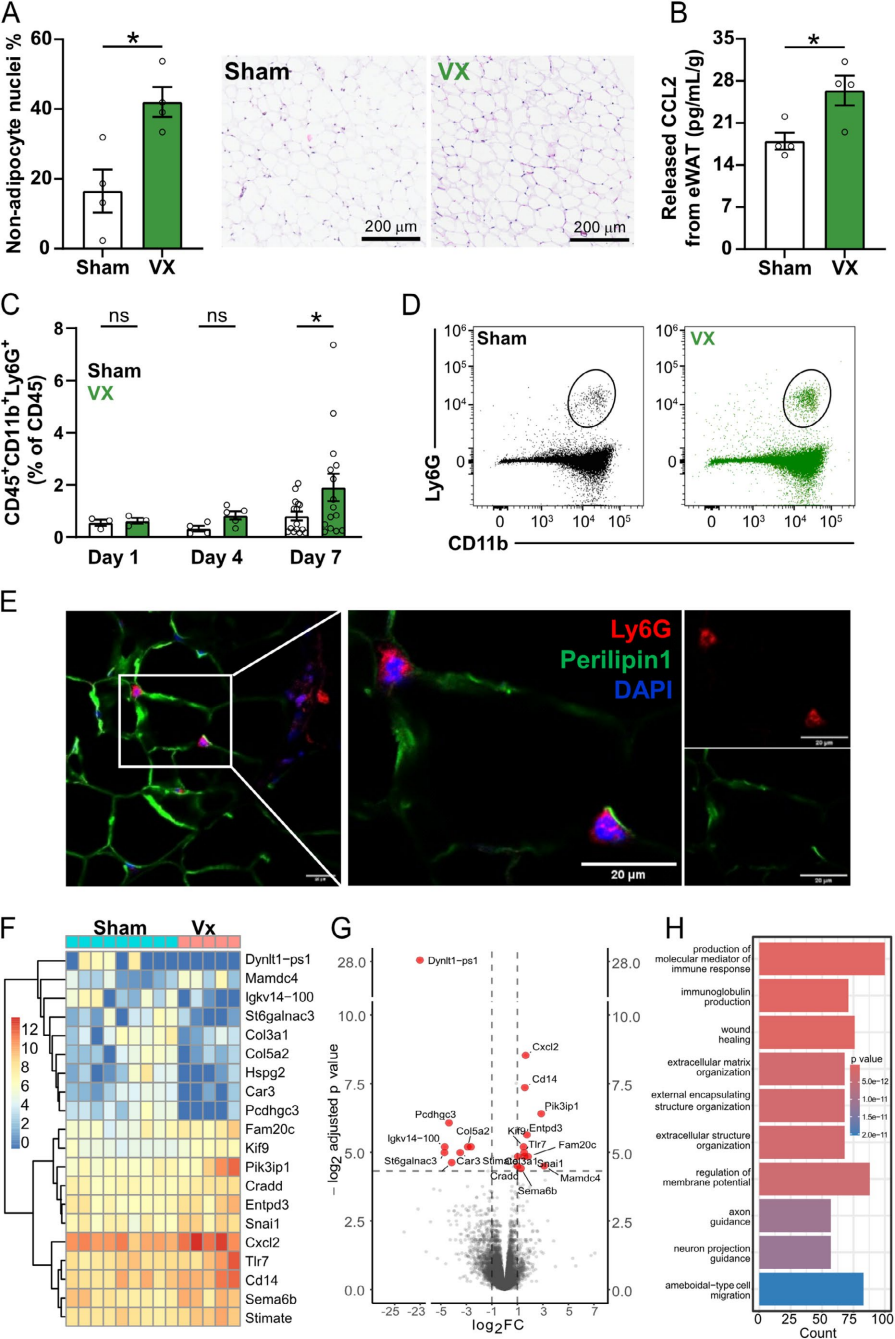
Vagotomy promoted Ly6G^+^ cell infiltration into eWAT. Wild‐type mice were subjected to left cervical vagotomy (VX) or sham surgery and tissues were collected at 7 days following surgery. (A) The percentage of non‐adipocyte nuclei of total nuclei per section was quantified using ImageJ (*n* = 4) and right panels show representative images of paraffin sections of eWAT stained with H&E in sham and VX animals. (B) CCL2 release from eWAT was analyzed by ELISA. The bar shows the CCL2 levels from sham (*n* = 4) or VX (*n* = 4) mice normalized to eWAT weight: ng/mL per g ± SEM (unpaired Student's *t* test). (C) eWAT was collected at 1 (*n* = 3), 4 (*n* = 4 sham, *n* = 5 VX), and 7 (*n* = 15) days following VX or sham surgery and the eWAT SVCs were analyzed by flow cytometry. The bar shows the % ± SEM of CD11b^+^Ly6G^+^ cells from CD45^+^ (one‐way ANOVA, Uncorrected Fisher's LSD). (D) Graphs show representative gating for CD11b^+^Ly6G^+^ cells in sham and VX eWAT at 7 days (concatenated *n* = 5–6). (E) Representative immunostaining of Ly6G (red) and Perilipin1 (green) in paraffin sections of eWAT. (F–H) Bone marrow neutrophils after sham (*n* = 9) or VX (*n* = 5) surgery were isolated using negative magnetic beads and analyzed using bulk RNAseq (DESeq2). Heatmap (F), volcano plot (G) of differentially expressed genes, and GO (Gene Ontology) (H) enrichment bar plot. ns = not significant, *p < 0.05. VX, Vagotomy; eWAT, epididymal white adipose tissue; H&E, hematoxylin–eosin; SVCs, stromal vascular cells.

Quantification of the nuclei ratio in the hematoxylin and eosin staining of eWAT sections showed a significantly higher non‐adipocyte nuclei percentage in eWAT following VX compared to sham surgery (Figure 2A, Figure S2B). Further, eWAT biopsies isolated from mice after VX released significantly higher levels of the immuno‐attractant CCL2 [34] per gram tissue as compared with eWAT isolated from sham‐treated mice (Figure 2B). Together, the observations support that VX is associated with increased eWAT immune cell infiltration.

We next investigated the composition of the eWAT stromal vascular cells (SVCs) following VX. No significant difference in eWAT proportion of CD45^+^, CD11b^−^Ly6G^−^CD19^+^CD3^−^, or CD11b^−^Ly6G^−^CD19^−^CD3^+^ (Figure S2C,D) was observed at 7 days following VX as compared with sham, or in the proportion of CD11b^+^Ly6G^−^F4/80^+^ at 1, 4, or 7 days following VX (Figure S2E). However, we observed a significantly higher proportion of CD11b^+^Ly6G^+^ cells (neutrophils) in eWAT at 7 days following VX as compared with sham surgery (Figure 2C,D). Immunohistochemistry showed that Ly6G^+^ cells were localized between Perilipin1^+^ adipocytes in eWAT (Figure 2E).

As these results indicate that VX promotes neutrophil infiltration into eWAT, we next investigated the role of VX on neutrophil identity in their primary reservoir, the bone marrow [35]. We isolated bone marrow neutrophils using negative selection beads at 7 days after VX of sham surgery. RNA‐sequencing revealed that levels of pro‐inflammatory and chemoattractant transcripts such as Tlr7, CD14, Cxcl2 were consistently upregulated following VX (Figure 2F,G). Gene ontology analysis of differentially expressed genes between VX‐ and sham‐treated mice indicated that neutrophil genes involved in immune responses, extracellular matrix organization, and migration were affected by vagotomy (Figure 2H). Collectively, these findings suggest that VX promotes a neutrophil phenotype characterized by enhanced pro‐inflammatory activity and migratory potential.

### 3.3 Genetic Deficiency of Ly6G ^+^ Cells Attenuated VX‐Associated Reduction of Body and eWAT Weight

**FIGURE 3:**
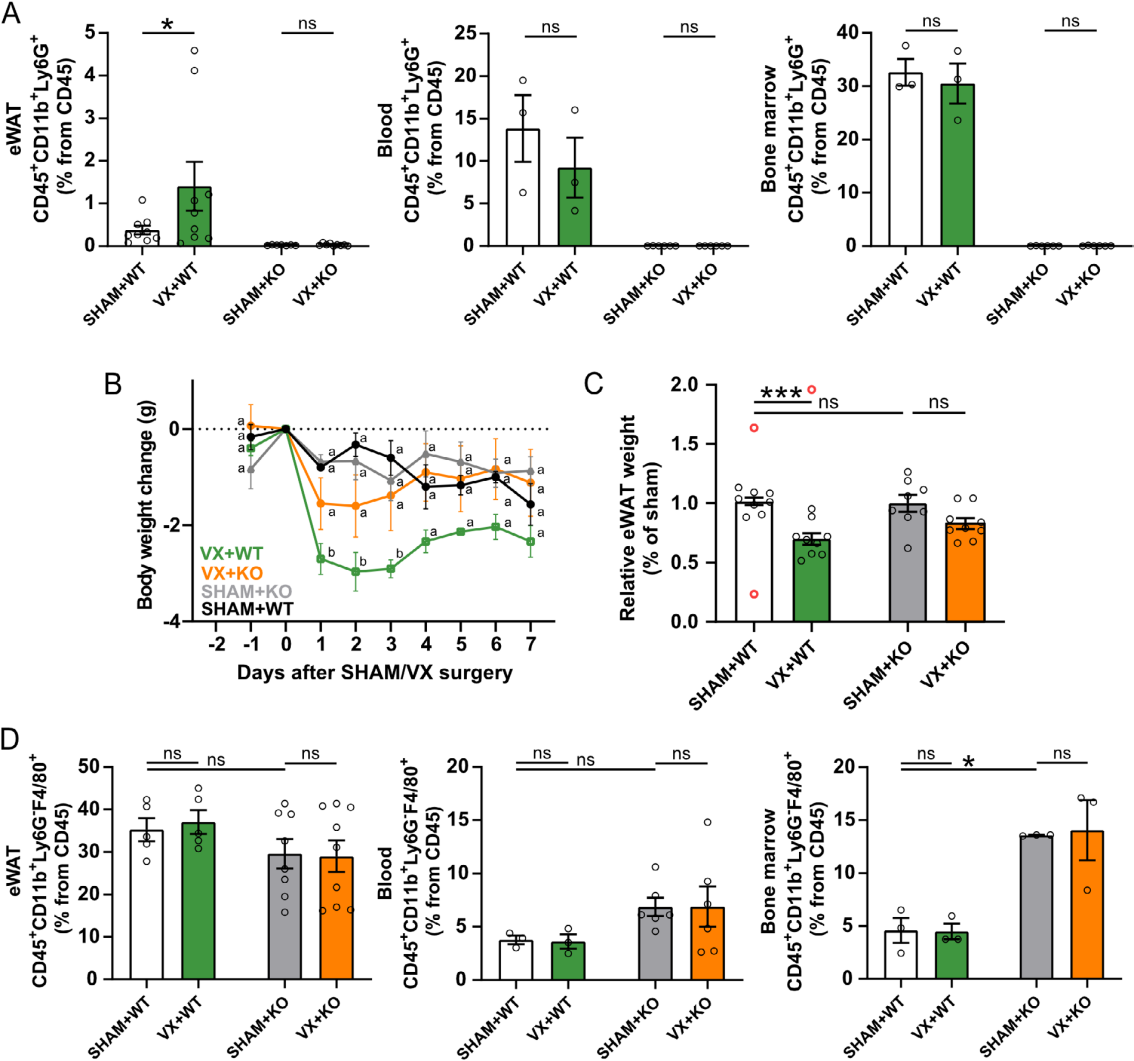
Neutrophil deficiency attenuated the VX‐associated reduction of eWAT weight. Ly6G^cre^Mcl1^fl/fl^ mice were subjected to VX or sham surgery and tissues were collected at 7 days following the surgery. (A) Flow cytometry analysis of CD11b^+^Ly6G^+^ cells in eWAT (*n* = 7–9), blood (*n* = 3–6), and bone marrow (*n* = 3–6) following VX or sham surgery in wild‐type (WT) and Ly6G^cre^Mcl1^fl/fl^ (KO) mice. Bars show the proportion of cells from CD45^+^ (one‐way ANOVA, uncorrected Fisher's LSD). (B) The graph shows the change in body weight of WT (*n* = 3) and Ly6G^cre^Mcl1^fl/fl^ (KO) (*n* = 3–6) mice shown as grams±SEM from body weight day 0 (two‐way ANOVA, Tukey's multiple comparisons test—Significant differences between experimental groups at each time point are indicated with a and b, and the detailed description can be found in Table S2). (C) eWAT weight was recorded at 7 days following VX or sham surgery (*n* = 8–10 sham, *n* = 9 VX). The bars depict the relative eWAT weight to sham eWAT weight in % ± SEM (one‐way ANOVA, Šídák's multiple comparisons test). (D) Flow cytometry analysis of CD11b^+^Ly6G^−^F4/80^+^ cells in eWAT (*n* = 5–8), blood (*n* = 3–6), and bone marrow (*n* = 3) following VX or sham surgery in WT and Ly6G^cre^Mcl1^fl/fl^ (KO) mice. Bars show the proportion of cells from CD45 (one‐way ANOVA, Šídák's multiple comparisons test). eWAT, epididymal white adipose tissue; ns, not significant, * *p* < 0.05, ***p < 0.001, VX, Vagotomy.

To investigate the role of neutrophils in VX‐associated weight and eWAT loss, we next performed VX or sham surgery in neutropenic Ly6G^cre^Mcl1^fl/fl^ mice. We confirmed that the mice lack Ly6G^+^ cells, including CD11b^+^Ly6G^+^ cells in eWAT, blood, and bone marrow, but have the same eWAT mass as wildtype mice (Figure 3A,C). Importantly, the significant reduction in body weight post‐VX was attenuated in the Ly6G^cre^Mcl1^fl/fl^ mice (Figure 3B). Similarly, there was no significant reduction in relative eWAT weight in Ly6G^cre^Mcl1^fl/fl^ mice at 7 days following VX as compared with sham‐treated animals (Figure 3C). These observations support the notion that neutrophils play a role in VX‐induced weight loss.

Subsequent analysis of Ly6G^cre^Mcl1^fl/fl^ mouse immune cells showed no VX‐mediated significant differences in CD11b^+^Ly6G^−^F4/80^+^ proportions in eWAT, blood, or bone marrow (Figure 3D), but did highlight that Ly6G^cre^Mcl^fl/fl^ mice have a significantly higher proportion of CD11b^+^Ly6G^−^F4/80^+^ cells in bone marrow as compared with wild‐type mice (Figure 3D). To study the mechanism further without this potential confounder, we turned to pharmacological blocking of Ly6G.

### 3.4 Extracellular Ly6G Ligation Attenuated VX‐Associated Reduction of Body Weight

**FIGURE 4:**
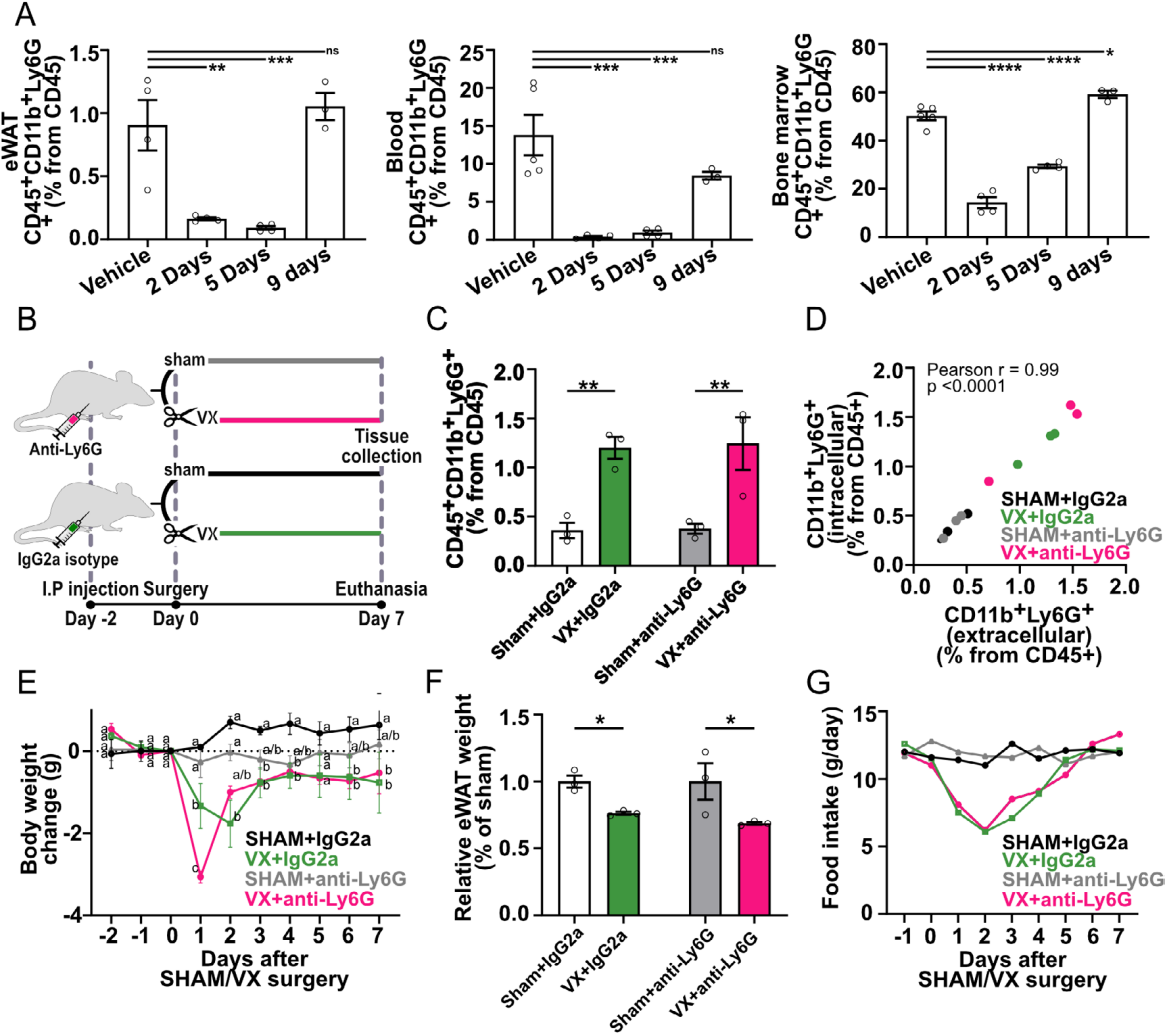
Temporary extracellular Ly6G depletion effect on VX‐associated reduction of eWAT weight. (A) Flow cytometry analysis of the frequency of extracellular Ly6G and CD11b double positive cells following one intraperitoneal injection of either anti‐Ly6G or vehicle (PBS). Bars show the % ± SEM of CD11b^+^Ly6G^+^ in vehicle (*n* = 4–5) and at 1 (*n* = 2), 2 (*n* = 4), 5 (*n* = 4), and 9 (*n* = 3) days following injection in eWAT, blood, and bone marrow (one‐way ANOVA, Šídák's multiple comparisons test). (B) Schematic diagram depicting the experimental setup: Wild‐type mice were intraperitoneally injected once with anti‐Ly6G antibody or IgG2a antibody 2 days before sham or VX surgery, and tissue was collected 7 days following surgery. (C) Flow cytometry analysis of CD11b^+^Ly6G^+^ cells in eWAT (*n* = 3) following VX or sham. Bars show the proportion of cells from CD45^+^ (one‐way ANOVA, uncorrected Fisher's LSD). (D) Correlation between extracellular and intracellular expression of Ly6G in flow cytometry analysis. Circles represent each sample stained for both extracellular and intracellular Ly6G in separate fluorescent channels (Pearson r correlation). (E) The mice were weighed daily. The graph shows the difference in body weight (g) of the mice from day 0 (before surgery) of each experimental group (*n* = 3) in g ± SEM (two‐way ANOVA, Tukey's multiple comparisons test—Significant differences between experimental groups at each time point are indicated with a, b, and c, and the detailed description can be found in Table S3). (F) eWAT weight (*n* = 3) was recorded at 7 days following VX or sham surgery. The bars show the relative eWAT weight to sham eWAT weight in % ± SEM (one‐way ANOVA, uncorrected Fisher's LSD). (G) Mice were kept in separate cages according to experimental groups: Sham+IgG2a, VX + IgG2a, sham+anti‐Ly6G, VX + anti‐Ly6G (*n* = 3). The food for each cage was weighed at the same time point daily. The curve shows the grams of food consumed per day per cage in g. ns = not significant, *p < 0.05, **p < 0.01, ***p < 0.001, ****p < 0.0001. VX, Vagotomy; eWAT, epididymal white adipose tissue.

To investigate the role of neutrophils and Ly6G in VX‐associated effect on eWAT in mice, we administered anti‐Ly6G antibodies in VX‐ and sham‐treated mice. Notably, administration of only anti‐Ly6G antibodies does not cause full neutropenia [36]. It ligates extracellular Ly6G, transiently masking the extracellular epitope and impairing neutrophil function. In mice without VX surgery, we confirmed that a single anti‐Ly6G injection significantly reduced the detection of extracellular Ly6G by flow cytometry in eWAT, blood, and bone marrow, and that the reduction was lost at 9 days after injection (Figure 4A). In this model of transient Ly6G‐ligation, VX promoted the previously observed significantly higher CD11b^+^Ly6G^+^ proportion in eWAT at 7 days post‐surgery in mice 9 days after one injection with both anti‐Ly6G or IgG2a control antibodies (Figure 4B,C) as compared with sham.

Further, validating that the Ly6G antibody block had receded day 7 post‐VX, we observed that extracellular Ly6G expression significantly correlated [*p* < 0.0001, Pearson r (rP) = 0.99] with intracellular (IN) Ly6G expression (Figure 4D) independent of antibody treatment [36]. Accordingly, in mice injected once with anti‐Ly6G or IgG2a antibodies, we observed a significant reduction in body weight (Figure 4E) and eWAT weight (Figure 4F) 7 days after VX as compared with sham. Administration of anti‐Ly6G or IgG2a control antibodies had no significant effect on measured food intake (Figure 4G). Consequently, a single anti‐Ly6G antibody injection promoted transient ligation of extracellular Ly6G without a significant effect on VX‐associated reduction of eWAT weight.

**FIGURE 5:**
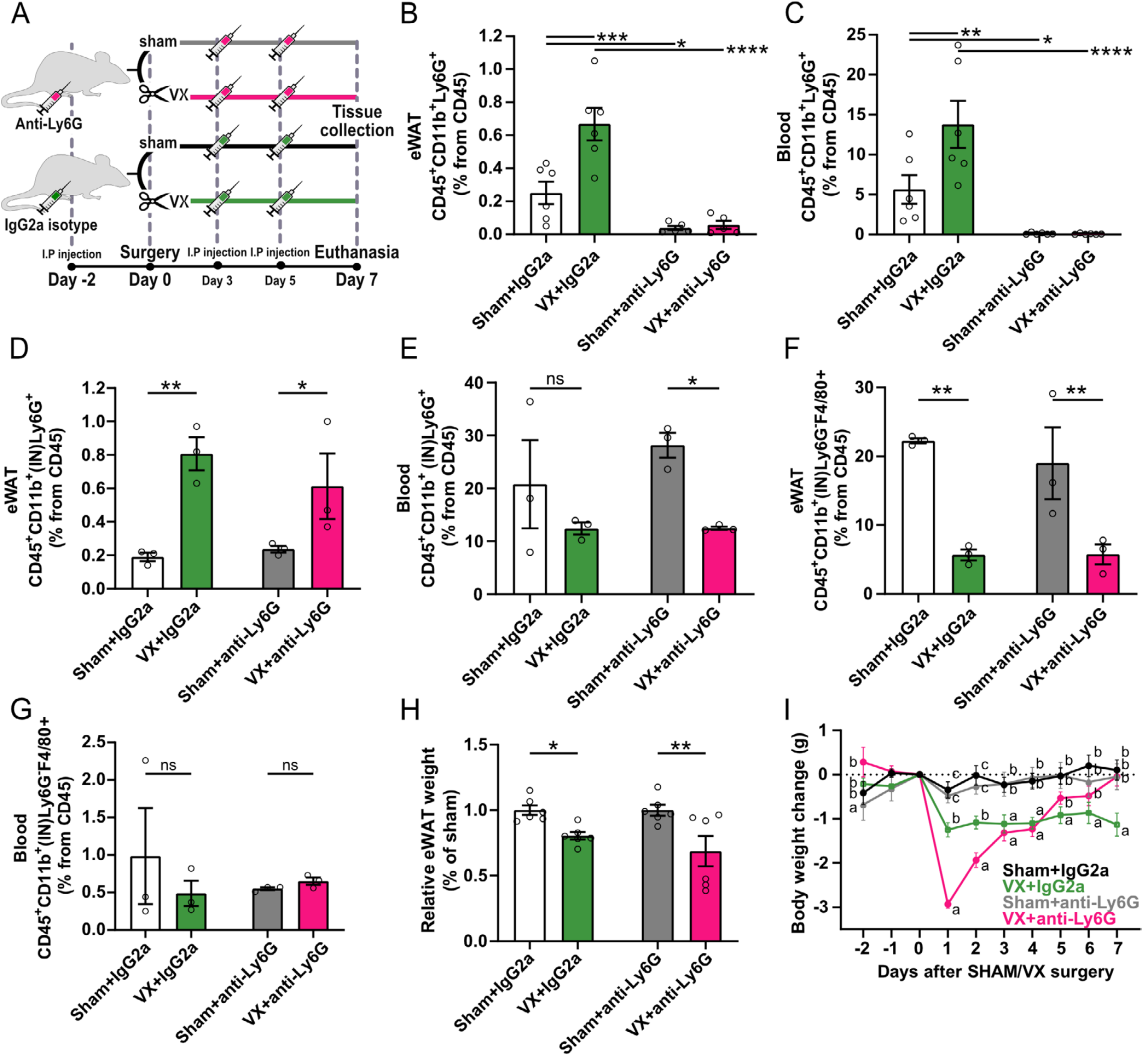
Extracellular Ly6G depletion attenuated VX‐mediated reduction of body weight. (A) Schematic of the experimental setup: Wild‐type mice were intraperitoneally injected with anti‐Ly6G antibody or IgG2a antibody 2 days before, as well as 3 and 5 days following sham or VX surgery, and tissue was collected 7 days following surgery. (B–C) Flow cytometry analysis of CD11b^+^Ly6G^+^ cells in eWAT (*n* = 6) and blood (*n* = 6) following VX or sham surgery, with IgG2a or anti‐Ly6G treatment. Bars show the proportion of cells from CD45^+^ (One‐way Anova, Uncorrected Fisher's LSD). (D–E) Flow cytometry analysis of intracellular Ly6G^+^ cells in eWAT (*n* = 3) and blood (*n* = 3) following VX or sham surgery, with IgG2a or anti‐Ly6G treatment. Bars show the proportion of cells from CD45^+^ (One‐way Anova, Uncorrected Fisher's LSD). (F–G) Flow cytometry analysis of CD11b^+^(IN)Ly6G^−^F4/80^+^ cells in eWAT (*n* = 3) and blood (*n* = 3) following VX or sham surgery, with IgG2a or anti‐Ly6G treatment. Bars show the proportion of cells from CD45^+^ (One‐way Anova, Uncorrected Fisher's LSD). (H) eWAT weight (*n* = 6) was recorded at 7 days following VX or sham surgery. The bars depict the relative eWAT weight to sham eWAT weight in % ± SEM (one‐way ANOVA, uncorrected Fisher's LSD). (I) The mice were weighed daily. The graph shows the difference in body weight of the mice from day 0 (before initiation of surgery) of each experimental group (*n* = 6) in % ± SEM (two‐way ANOVA, Tukey's multiple comparisons test—Significant differences between experimental groups at each time point are indicated with a, b, and c, and the detailed description can be found in Table S4). ns = not significant, *p < 0.05, **p < 0.01, ***p < 0.001, ****p < 0.0001. VX, Vagotomy; eWAT, epididymal white adipose tissue; BM, bone marrow; SVCs, stromal vascular cells; intracellular (IN).

To achieve sustained extracellular Ly6G ligation, mice were injected intraperitoneally with anti‐Ly6G antibodies at −2, +3, and +5 days relative to the time of surgery (Figure 5A). Extracellular Ly6G staining was significantly lower in both eWAT and blood following anti‐Ly6G injection at 7 days following sham and VX surgery (Figure 5B,C). Interestingly, in addition to the expected significantly higher proportion of CD11b^+^Ly6G^+^ (neutrophil) cells in eWAT at 7 days following VX (Figure 5B), we observed a significantly higher proportion of CD11b^+^Ly6G^+^ cells in blood following VX in the IgG2a control animals (Figure 5C).

Next, we utilized the intracellular (IN) expression of Ly6G to identify Ly6G‐positive and Ly6G‐negative cells in this experimental model. As expected, we observed a significantly higher proportion of eWAT CD11b^+^(IN)Ly6G^+^ cells following VX in both IgG2a and anti‐Ly6G treated mice, which did not differ between the antibody treatments (Figure 5D, Figure S3A). Furthermore, there was a significantly lower proportion of CD11b^+^(IN)Ly6G^+^ cells in the blood of mice that received anti‐Ly6G injections following VX as compared to sham (Figure 5E). Further investigation revealed a significantly lower proportion of eWAT CD11b^+^(IN)Ly6G^−^F4/80^+^ cells following VX, as compared with sham, in both IgG2a and anti‐Ly6G treated mice (Figure 5F, Figure S3B). No significant differences were observed in the proportion of CD11b^+^(IN)Ly6G^−^F4/80^+^ cells in the blood, suggesting this to be a WAT‐specific phenotype (Figure 5G).

Next, we investigated body and eWAT weight following VX or sham surgery and sustained ligation of Ly6G. We observed a significant VX‐associated reduction in relative eWAT weight in both the IgG2a‐ and anti–Ly6G‐treated mice as compared with sham at day 7 post‐surgery (Figure 5H). Interestingly, the body weight of the VX + anti‐Ly6G‐treated mice returned to baseline by day 7, whereas the VX + IgG2a mouse weight remained significantly lower (Figure 5I). Furthermore, anti‐Ly6G treatment promoted a significantly greater reduction in body weight 1 day after VX surgery as compared with VX + IgG2a (Figure 5I), without an anti–Ly6G‐mediated effect on food intake, which had approximately equalized between all groups by day 5 (Figure S3C). Together, these findings suggest that extracellular Ly6G expression contributes to the VX‐associated sustained reduction in mouse body weight.

## 4 Discussion

In this study, we found that unilateral cervical vagotomy induces weight loss and promotes in vitro release of lipids from epididymal white adipose tissue (eWAT) under conditions without induced inflammation. This metabolic change was accompanied by infiltration of Ly6G^+^ cells into eWAT. Ligation of the neutrophil differentiation antigen Ly6G by antibody injection abolished the VX‐associated weight loss. The data highlights a previously unexplored role for Ly6G in early temporal regulation of adipose tissue physiology following vagotomy.

Consistent with previous observations [11], unilateral vagotomy led to a reduction in both body weight and eWAT mass 1 week post‐surgery, along with a significant decrease in adipocyte area. Although vagus nerve signaling is well‐established in the regulation of short‐term feeding behavior [37], and we observed transient reductions in food intake following vagotomy, the normalization of intake by day five suggests that the sustained weight loss may not be attributed solely to decreased appetite. Lipid storage in adipocytes is mainly determined by the balance of lipolysis and lipogenesis, processes regulated by hormonal and nutritional cues [38]. Our data support a model in which VX promotes lipolysis, as reflected by the increased NEFA release from eWAT in vagotomized mice.

The elevated levels of CCL2 in eWAT of vagotomized mice, compared with sham‐operated controls, suggest a shift toward a more pro‐inflammatory adipose tissue phenotype following vagotomy. The data are consistent with previous reports on increased inflammation and impaired resolution of inflammation after vagotomy [39, 40, 41, 42]. These notions are also in line with the gene expression analysis of bone marrow: neutrophils from vagotomized mice showed an enrichment of transcripts related to mediators of immune responses. Together, the data here indicate that vagotomy promoted a neutrophile phenotype more prone to pro‐inflammatory responses [43, 44, 45], observations that further support a homeostatic role for the vagus nerve in inflammation. However, it also suggests that organ‐specific immune responses outside adipose tissue cannot be fully excluded and warrant more systematic evaluation in future work.

While CD45^+^CD11b^+^F4/80^+^ proportions in eWAT remained unchanged across most experimental conditions, we observed a significant VX‐mediated difference in the proportion of these cells following Ly6G manipulation. These findings suggest that CD45^+^CD11b^+^F4/80^+^ may not regulate lipolytic activity in this specific VX‐associated context. The role of macrophages in adipose tissue function is however well established, and it cannot be excluded that aspects of their phenotype, that were not investigated here, were changed following vagotomy. This should be addressed in future studies.

Importantly, we observed a significant increase in CD45^+^CD11b^+^Ly6G^+^ cell proportion in eWAT 1 week after VX, with these cells localized in between adipocytes. Neutrophils are traditionally associated with acute inflammation and antimicrobial defense [46], but emerging evidence suggests they may also play roles in metabolic regulation [47]. Lipolysis is known to recruit leukocytes into adipose tissue [21], and inflammation can trigger lipolysis [48]. The observation that genetic ablation of Ly6G^+^ cells abrogated VX‐associated weight loss implicates a role for neutrophils as regulators of lipolysis in this context.

Interestingly, antibody‐mediated ligation of extracellular Ly6G which functionally blocks neutrophils, also attenuated VX‐associated weight loss. This suggests that Ly6G itself may play a role in VX‐mediated lipolysis. The persistence of intracellular Ly6G expression following antibody treatment confirms the presence of neutrophils, but with ligated surface Ly6G. This important difference between the genetic ablation and the antibody‐mediated Ly6G‐ligation may explain the differences in the observations between the different experimental approaches used here. Given that Ly6G ligation can impair neutrophil migration [24, 49], and that we did not observe a reduction in eWAT neutrophil proportions following Ly6G ligation, it is possible that neutrophils utilize another Ly6G/beta‐2 integrin independent migration mechanism [23, 50] into eWAT following vagotomy in this setting. The exact cues governing neutrophil migration into eWAT following vagotomy should be addressed further in future studies to elucidate the systemic versus local, and potentially also right versus left, vagal effect on neutrophil behavior in this context.

Taken together, our data reveal a previously unrecognized role for Ly6G in regulating adipose tissue homeostasis and body weight following vagotomy. These observations highlight the involvement of Ly6G in the regulation of metabolism in the context of vagotomy and warrant further investigation.

## Author Contributions

Conceptualization; A.S.C., T.L., C.E.H., P.S.O., and L.T. Funding acquisition and project administration; C.E.H., P.S.O., and L.T. Investigation and validation; T.L., A.S.C., M.C., P.M.T., Q.G., R.R.S., W.D., J.J.V., P.H., M.V., P.T.M., V.S.S., and L.T. Methodology; T.L., A.S.C., M.C., and L.T. Resources; C.E.H., M.C.I.K., and P.S.O. Formal analysis; M.C., M.V., and V.S.S. Software; M.C. Supervision; S.G.M., C.E.H., P.S.O., and L.T. Visualization; T.L. and L.T. Writing – original draft; T.L., V.S.S., C.E.H., P.S.O., and L.T. Writing – review and editing; A.S.C., S.G.M., M.C.I.K., V.S.S., C.E.H., F.W., P.S.O., and L.T.

## Funding

This study was supported by grants from the NovoNordisk post‐doctoral program, NovoNordisk Foundation project grant (CH: 0092494), and the Swedish Research Council (C.E.H: 2024–02432) (P.S.O.: 2020–04443, 2024–03735), Heart‐Lung Foundation (P.S.O.: 20200827, 20210524, 20230006), Karolinska Institutet (P.S.O.: 2023–01461, FoUI‐987284) and MedTechLabs to P.S.O. T.L., Q.G., and M.C. were supported in part by the KI‐CSC (China Scholarship Council) program at Karolinska Institutet (MC: 202206380036), L.T. was supported by Karolinska Institutet (2022–0170).

## Conflicts of Interest

P.S.O. is a shareholder of Emune AB. The other authors have no conflicts of interest.

## Supporting information


**Appendix S1:** (A) Body weight (g) before (day 0) and 1 day after sham (*n* = 3) or VX (*n* = 3) surgery. Each mouse is represented by two dots connected by a line (Paired Student's *t* test). (B) Body weight (g) before (day 0) and 4 days after sham (*n* = 4) or VX (*n* = 5) surgery. Each mouse is represented by two dots connected by a line (Paired Student's *t* test). (C) Body weight (g) before (day 0) and 7 days sham surgery (*n* = 19) or VX (*n* = 21). Each mouse is represented by two dots connected by a line (Paired Student's *t* test). (D‐E) Representative images of the quantification of adipocyte cell size using image J in sections of eWAT collected 7 days after sham (D) or VX (E) stained with BODIPY FL C12.
**Figure S2:**. (A) Blood, bone marrow, and spleens were collected at 7 days following VX or sham surgery and CD11b^+^Ly6G^+^, CD11b^+^Ly6G^−^F4/80^+^, CD11b^−^Ly6G^−^CD19^+^CD3^−^, and CD11b^−^Ly6G^−^CD19^−^CD3^+^ proportions were analyzed by flow cytometry. The bar shows the %±SEM of cells from CD45^+^ (Unpaired Student's *t* test), n is indicated in each figure panel. (B) Representative eWAT gating strategy for SVF analysis (1:1) and for CD 45% analysis (1:2). (C‐D) eWAT were collected at 7 days following VX or sham surgery and the SVCs were analyzed for (C) CD45^+^ or (D) CD11b^−^Ly6G^−^CD19^+^CD3^−^, and CD11b^−^Ly6G^−^CD19^−^CD3^+^ proportion. The bar shows the %±SEM of cells (unpaired Student's *t* test), n is indicated in each figure panel. Lower D panel shows representative gating for CD3 and CD19 in sham and VX eWAT (concatenated *n* = 5‐6). (E) eWAT was collected at 1 (*n* = 3), 4 (*n* = 4 sham, *n* = 5 VX), and 7 (*n* = 8) days following VX or sham surgery and the eWAT SVCs were analyzed by flow cytometry. The bar shows the %±SEM of CD11b^+^Ly6G^−^F4/80^+^ cells from CD45^+^ (One‐way ANOVA, Uncorrected Fisher's LSD). Right panels show representative gating for CD11b and F4/80 in sham and VX eWAT at 7 days (concatenated *n* = 5‐6).
**Figure S3:** Concatenated representative gating in eWAT following VX or sham surgery, with IgG2a or anti‐Ly6G treatment. (A) Single, alive, CD45^+^, intracellular (IN) Ly6G^+^CD11b^+^ cells. (B) Single, alive, CD45^+^, intracellular (IN) Ly6G negative, F480^+^, and CD11b^+^ cells. (C) Mice were kept in separate cages according to experimental groups: sham+IgG2a, VX+IgG2a, sham+anti‐Ly6G, VX+anti‐Ly6G (*n* = 3). The food for each cage was weighed at the same time daily. The curve shows the grams of food consumed per day per cage in g.
**Table S1:** Antibodies used for flow cytometry.
**Table S2:** Tukey's multiple comparisons test for the weight of wild type (WT) and Ly6G^cre^Mcl1^fl/fl^ (KO) mice following vagotomy (VX) or sham surgery.
**Table S3:** Tukey's multiple comparisons test weight of mice following single extracellular Ly6G ligation (anti‐Ly6G), isotype control (IgG2a), and vagotomy (VX) or sham surgery.
**Table S4:** Tukey's multiple comparisons test weight of mice following sustained extracellular Ly6G ligation (anti‐Ly6G), isotype control (IgG2a), and vagotomy (VX) or sham surgery.

## Data Availability

The data that support the findings of this study are available on request from the corresponding author.
