# Supplementary material for: Lymphocyte Antigen 6G Mediates Vagotomy‐Associated Reduction in Body Weight
Source: FASEB J. 2026 Apr 4;40(7):e71716. doi: 10.1096/fj.202600151RR (PMC13050019; doi:10.1096/fj.202600151RR)
Supplement: Supplementary file 1 — Appendix S1: (A) Body weight (g) before (day 0) and 1 day after sham (n = 3) or VX (n = 3) surgery. Each mouse is represented by two dots connected by a line (Paired Student's t test). (B) Body weight (g) before (day 0) and 4 days after sham (n = 4) or VX (n = 5) surgery. Each mouse is represented by two dots connected by a line (Paired Student's t test). (C) Body weight (g) before (day 0) and 7 days sham surgery (n = 19) or VX (n = 21). Each mouse is represented by two dots connected by a line (Paired Student's t test). (D‐E) Representative images of the quantification of adipocyte cell size using image J in sections of eWAT collected 7 days after sham (D) or VX (E) stained with BODIPY FL C12. Figure S2:. (A) Blood, bone marrow, and spleens were collected at 7 days following VX or sham surgery and CD11b+Ly6G+, CD11b+Ly6G−F4/80+, CD11b−Ly6G−CD19+CD3−, and CD11b−Ly6G−CD19−CD3+ proportions were analyzed by flow cytometry. The bar shows the %±SEM of cells from CD45+ (Unpaired Student's t test), n is indicated in each figure panel. (B) Representative eWAT gating strategy for SVF analysis (1:1) and for CD 45% analysis (1:2). (C‐D) eWAT were collected at 7 days following VX or sham surgery and the SVCs were analyzed for (C) CD45+ or (D) CD11b−Ly6G−CD19+CD3−, and CD11b−Ly6G−CD19−CD3+ proportion. The bar shows the %±SEM of cells (unpaired Student's t test), n is indicated in each figure panel. Lower D panel shows representative gating for CD3 and CD19 in sham and VX eWAT (concatenated n = 5‐6). (E) eWAT was collected at 1 (n = 3), 4 (n = 4 sham, n = 5 VX), and 7 (n = 8) days following VX or sham surgery and the eWAT SVCs were analyzed by flow cytometry. The bar shows the %±SEM of CD11b+Ly6G−F4/80+ cells from CD45+ (One‐way ANOVA, Uncorrected Fisher's LSD). Right panels show representative gating for CD11b and F4/80 in sham and VX eWAT at 7 days (concatenated n = 5‐6). Figure S3: Concatenated representative gating in eWAT following VX or sham surgery, with IgG2a or anti‐Ly6G [file FSB2-40-e71716-s001.docx]

Supplemental Information


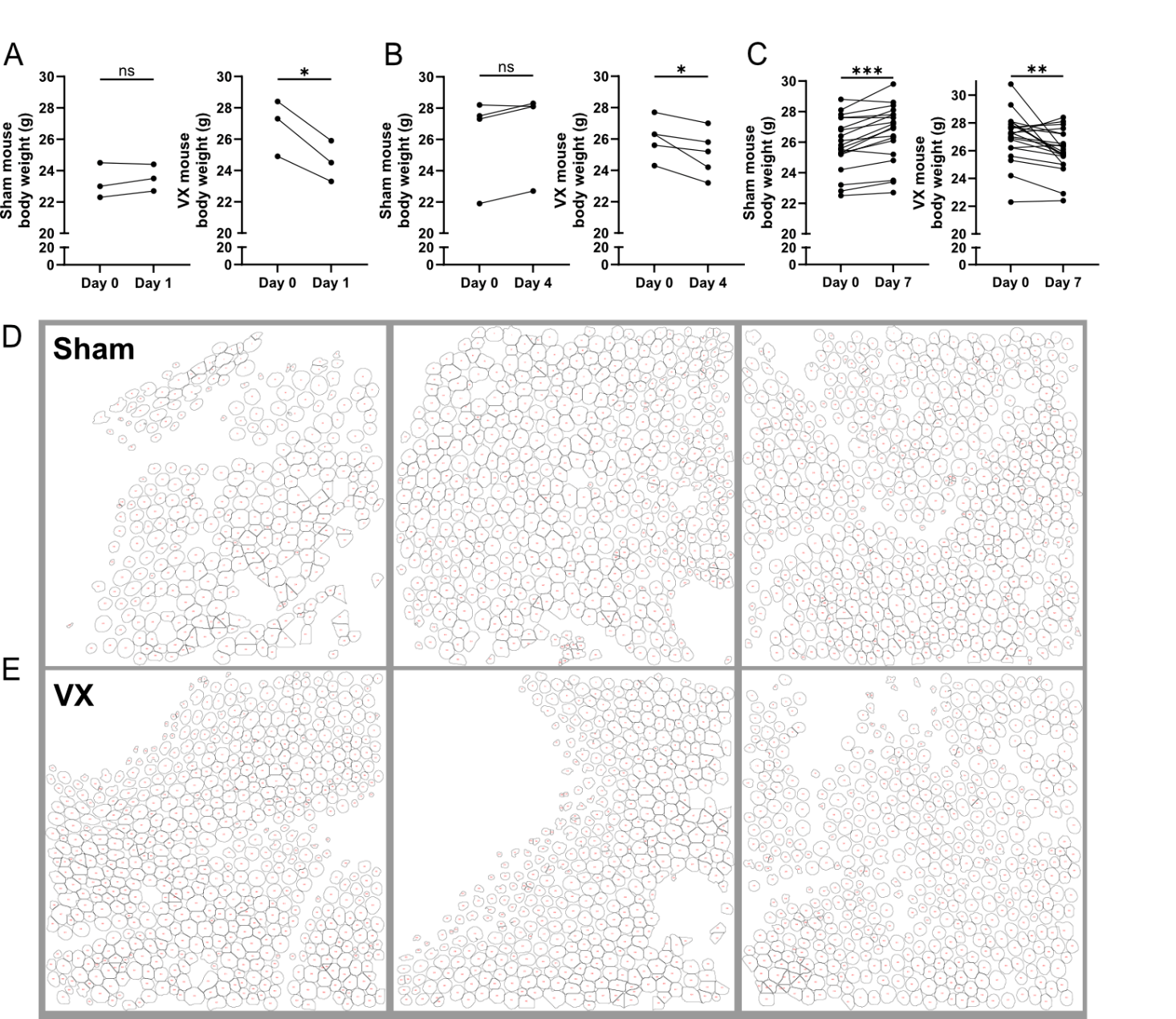


**Figure S1.** **(A)** Body weight (g) before (day 0) and 1 day after sham (n=3) or VX (n=3) surgery. Each mouse is represented by two dots connected by a line (Paired Student’s *t* test). **(B)** Body weight (g) before (day 0) and 4 days after sham (n=4) or VX (n=5) surgery. Each mouse is represented by two dots connected by a line (Paired Student’s *t* test). **(C)** Body weight (g) before (day 0) and 7 days sham surgery (n=19) or VX (n=21). Each mouse is represented by two dots connected by a line (Paired Student’s *t* test). **(D-E)** Representative images of the quantification of adipocyte cell size using image J in sections of eWAT collected 7 days after sham **(D)** or VX **(E)** stained with BODIPY™ FL C12.


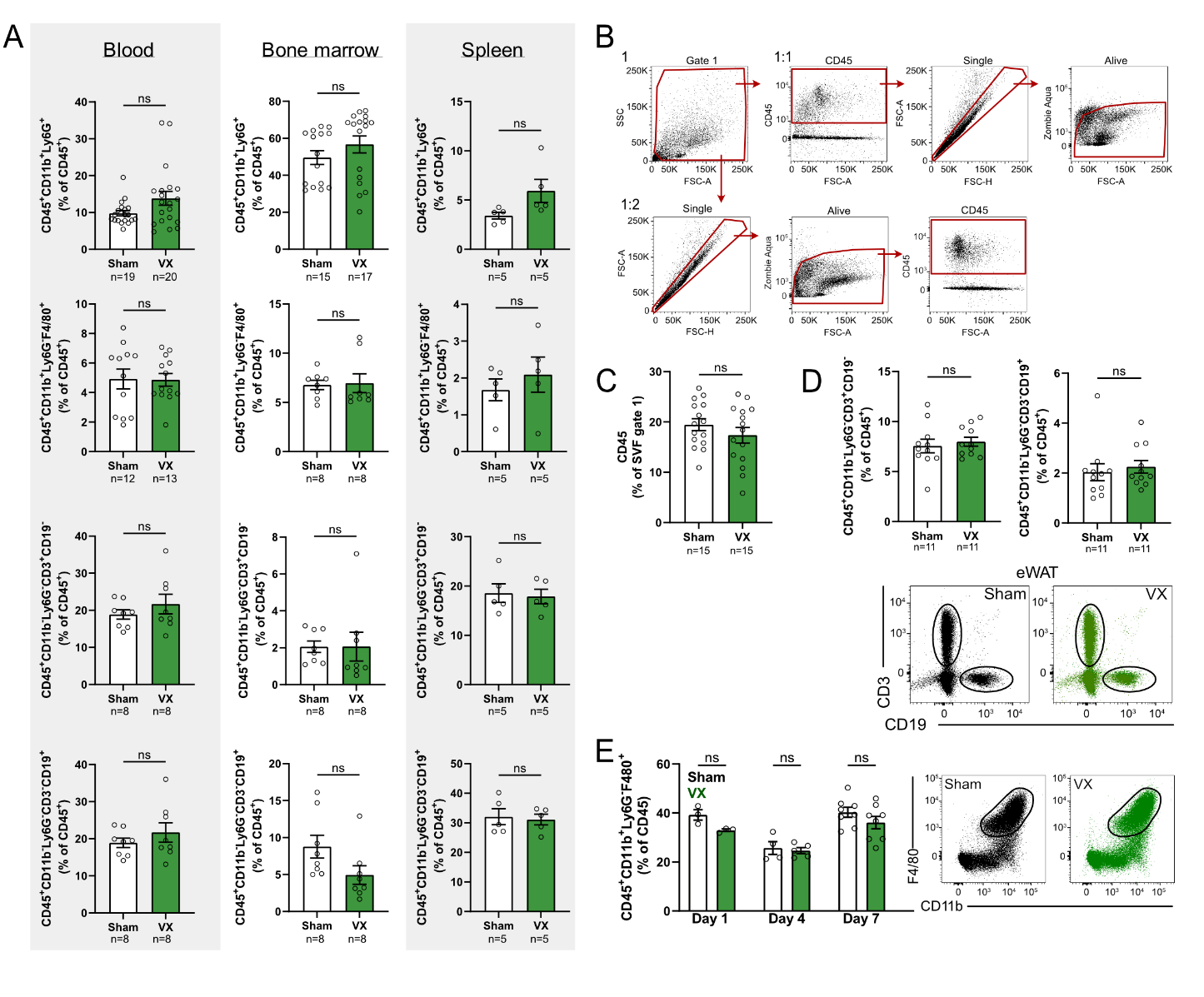


**Figure S2**. **(A)** Blood, bone marrow, and spleens were collected at 7 days following VX or sham surgery and CD11b^+^Ly6G^+^, CD11b^+^Ly6G^-^F4/80^+^, CD11b^-^Ly6G^-^CD19^+^CD3^-^, and CD11b^-^Ly6G^-^CD19^-^CD3^+^ proportions were analyzed by flow cytometry. The bar shows the %±SEM of cells from CD45^+^ (Unpaired Student’s *t* test), n is indicated in each figure panel. **(B)** Representative eWAT gating strategy for SVF analysis (1:1) and for CD45 % analysis (1:2). **(C-D)** eWAT were collected at 7 days following VX or sham surgery and the SVCs were analyzed for **(C)** CD45^+^ or **(D)** CD11b^-^Ly6G^-^CD19^+^CD3^-^, and CD11b^-^Ly6G^-^CD19^-^CD3^+^ proportion. The bar shows the %±SEM of cells (Unpaired Student’s *t* test), n is indicated in each figure panel. Lower D panel show representative gating for CD3 and CD19 in sham and VX eWAT (concatenated n=5-6). **(E)** eWAT was collected at 1 (n=3), 4 (n=4 sham, n=5 VX), and 7 (n=8) days following VX or sham surgery and the eWAT SVCs were analyzed by flow cytometry. The bar shows the %±SEM of CD11b^+^Ly6G^-^F4/80^+^ cells from CD45^+^ (One-way ANOVA, Uncorrected Fisher's LSD). Right panels show representative gating for CD11b and F4/80 in sham and VX eWAT at 7 days (concatenated n=5-6).


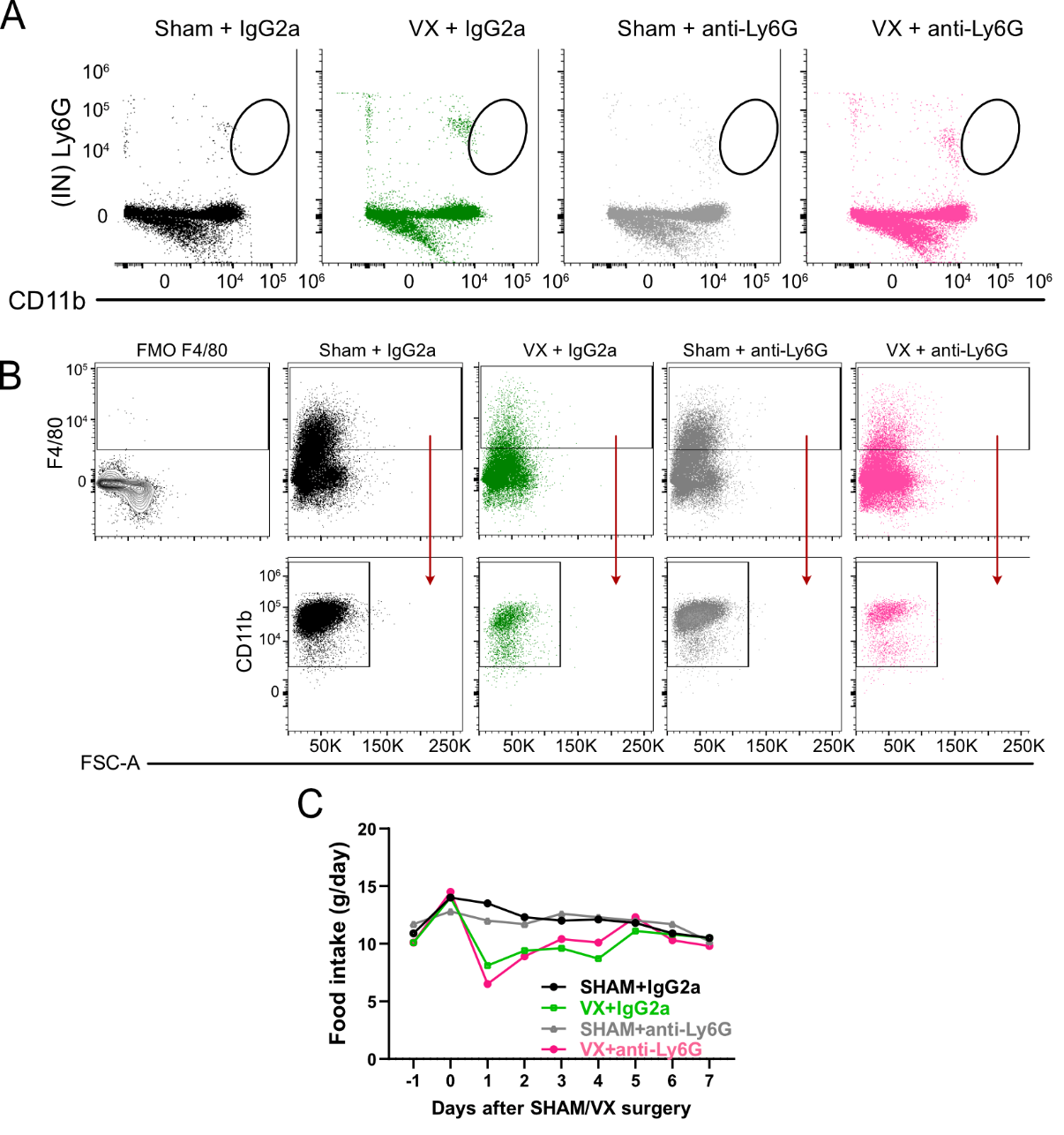


**Figure S3:** Concatenated representative gating in eWAT following VX or sham surgery, with IgG2a or anti-Ly6G treatment. **(A)** Single, alive, CD45^+^, intracellular (IN) Ly6G^+^CD11b^+^ cells. **(B)** Single, alive, CD45^+^, intracellular (IN) Ly6G negative, F480^+^ and CD11b^+^ cells. **(C)** Mice were kept in separate cages according to experimental groups: sham+IgG2a, VX+IgG2a, sham+anti-Ly6G, VX+anti-Ly6G (n=3). The food for each cage was weighed at the same time daily. The curve shows the grams of food consumed per day per cage in g.

**Table S1.** Antibodies used for flow cytometry.

| Antibodies | Company | Product# |
| --- | --- | --- |
| Zombie Aqua Fixable Viability Kit | BioLegend | 423101 |
| CD45-AF700 | Invitrogen | 56-0451-82 |
| CD11b-PE-CY7 | Invitrogen | 25-0112-82 |
| CD11b-APC | invitrogen | 17-0112-81 |
| Ly6G-BV421 | BioLegend | 127627 |
| Ly6G-FITC | BioLegend | 127605 |
| Ly6G-APC | BioLegend | 127613 |
| CD3-BV421 | BioLegend | 100335 |
| CD3e-PE | Invitrogen | 145-2C11 |
| CD3-APC-CY7 | BioLegend | 100221 |
| CD19-FITC | invitrogen | 11-0193-85 |
| Ly6C-BV785 | BioLegend | 128041 |
| F480-BV711 | BD Horizon | 565612 |

**Table S2.** Tukey's multiple comparisons test for weight of wild type (WT) and Ly6G^cre^Mcl1^fl/fl^ (KO) mice following vagotomy (VX) or sham surgery.

| Tukey's multiple comparisons test | Mean diff. | 95.00% CI of diff. | Summary | Adjusted P Value |
| --- | --- | --- | --- | --- |
|  |  |  |  |  |
| Day -1 |  |  |  |  |
| SHAM+WT vs. VX+WT | 0.23 | -1.94 to 2.41 | ns | 0.992 |
| SHAM+WT vs. SHAM+KO | 0.67 | -1.51 to 2.84 | ns | 0.855 |
| SHAM+WT vs. VX+KO | -0.23 | -2.41 to 1.94 | ns | 0.992 |
| VX+WT vs. SHAM+KO | 0.43 | -1.74 to 2.61 | ns | 0.954 |
| VX+WT vs. VX+KO | -0.47 | -2.64 to 1.71 | ns | 0.944 |
| SHAM+KO vs. VX+KO | -0.90 | -3.08 to 1.28 | ns | 0.704 |
|  |  |  |  |  |
| Day 0 |  |  |  |  |
| SHAM+WT vs. VX+WT | -6.66E-16 | -2.18 to 2.18 | ns | >0.999 |
| SHAM+WT vs. SHAM+KO | -8.88E-16 | -1.89 to 1.89 | ns | >0.999 |
| SHAM+WT vs. VX+KO | 0 | -1.89 to 1.89 | ns | >0.999 |
| VX+WT vs. SHAM+KO | -2.22E-16 | -1.89 to 1.89 | ns | >0.999 |
| VX+WT vs. VX+KO | 6.66E-16 | -1.89 to 1.89 | ns | >0.999 |
| SHAM+KO vs. VX+KO | 8.88E-16 | -1.54 to 1.54 | ns | >0.999 |
|  |  |  |  |  |
| Day 1 |  |  |  |  |
| SHAM+WT vs. VX+WT | 1.90 | -0.28 to 4.08 | ns | 0.109 |
| SHAM+WT vs. SHAM+KO | -0.12 | -2.00 to 1.77 | ns | 0.999 |
| SHAM+WT vs. VX+KO | 0.75 | -1.14 to 2.64 | ns | 0.728 |
| VX+WT vs. SHAM+KO | -2.02 | -3.90 to -0.13 | * | 0.031 |
| VX+WT vs. VX+KO | -1.15 | -3.04 to 0.74 | ns | 0.388 |
| SHAM+KO vs. VX+KO | 0.87 | -0.67 to 2.40 | ns | 0.460 |
|  |  |  |  |  |
| Day 2 |  |  |  |  |
| SHAM+WT vs. VX+WT | 2.64 | 0.46 to 4.82 | * | 0.011 |
| SHAM+WT vs. SHAM+KO | 0.34 | -1.54 to 2.23 | ns | 0.965 |
| SHAM+WT vs. VX+KO | 1.28 | -0.61 to 3.16 | ns | 0.297 |
| VX+WT vs. SHAM+KO | -2.30 | -4.18 to -0.41 | * | 0.010 |
| VX+WT vs. VX+KO | -1.37 | -3.25 to 0.52 | ns | 0.239 |
| SHAM+KO vs. VX+KO | 0.93 | -0.61 to 2.47 | ns | 0.394 |
|  |  |  |  |  |
| Day 3 |  |  |  |  |
| SHAM+WT vs. VX+WT | 2.30 | 0.13 to 4.48 | * | 0.034 |
| SHAM+WT vs. SHAM+KO | 0.47 | -1.41 to 2.36 | ns | 0.916 |
| SHAM+WT vs. VX+KO | 0.78 | -1.11 to 2.67 | ns | 0.703 |
| VX+WT vs. SHAM+KO | -1.83 | -3.72 to 0.05 | ns | 0.059 |
| VX+WT vs. VX+KO | -1.52 | -3.41 to 0.36 | ns | 0.157 |
| SHAM+KO vs. VX+KO | 0.31 | -1.23 to 1.85 | ns | 0.953 |
|  |  |  |  |  |
| Day 4 |  |  |  |  |
| SHAM+WT vs. VX+WT | 1.13 | -1.04 to 3.31 | ns | 0.529 |
| SHAM+WT vs. SHAM+KO | -0.68 | -2.57 to 1.20 | ns | 0.781 |
| SHAM+WT vs. VX+KO | -0.30 | -2.19 to 1.59 | ns | 0.976 |
| VX+WT vs. SHAM+KO | -1.82 | -3.70 to 0.07 | ns | 0.063 |
| VX+WT vs. VX+KO | -1.43 | -3.32 to 0.45 | ns | 0.201 |
| SHAM+KO vs. VX+KO | 0.38 | -1.16 to 1.92 | ns | 0.916 |
|  |  |  |  |  |
| Day 5 |  |  |  |  |
| SHAM+WT vs. VX+WT | 0.97 | -1.21 to 3.14 | ns | 0.655 |
| SHAM+WT vs. SHAM+KO | -0.48 | -2.37 to 1.40 | ns | 0.909 |
| SHAM+WT vs. VX+KO | -0.13 | -2.02 to 1.75 | ns | 0.998 |
| VX+WT vs. SHAM+KO | -1.45 | -3.34 to 0.44 | ns | 0.192 |
| VX+WT vs. VX+KO | -1.10 | -2.99 to 0.79 | ns | 0.428 |
| SHAM+KO vs. VX+KO | 0.35 | -1.19 to 1.89 | ns | 0.934 |
|  |  |  |  |  |
| Day 6 |  |  |  |  |
| SHAM+WT vs. VX+WT | 1.03 | -1.14 to 3.21 | ns | 0.605 |
| SHAM+WT vs. SHAM+KO | -0.08 | -1.97 to 1.80 | ns | 0.996 |
| SHAM+WT vs. VX+KO | -0.17 | -2.05 to 1.72 | ns | 0.996 |
| VX+WT vs. SHAM+KO | -1.12 | -3.00 to 0.77 | ns | 0.415 |
| VX+WT vs. VX+KO | -1.20 | -3.09 to 0.69 | ns | 0.350 |
| SHAM+KO vs. VX+KO | -0.08 | -1.62 to 1.46 | ns | 0.999 |
|  |  |  |  |  |
| Day 7 |  |  |  |  |
| SHAM+WT vs. VX+WT | 0.77 | -1.41 to 2.94 | ns | 0.795 |
| SHAM+WT vs. SHAM+KO | -0.70 | -2.59 to 1.19 | ns | 0.768 |
| SHAM+WT vs. VX+KO | -0.45 | -2.34 to 1.44 | ns | 0.925 |
| VX+WT vs. SHAM+KO | -1.47 | -3.35 to 0.42 | ns | 0.184 |
| VX+WT vs. VX+KO | -1.22 | -3.10 to 0.67 | ns | 0.338 |
| SHAM+KO vs. VX+KO | 0.25 | -1.29 to 1.79 | ns | 0.975 |

**Table S3.** Tukey's multiple comparisons test weight of mice following single extracellular Ly6G ligation (anti-Ly6G), isotype control (IgG2a), and vagotomy (VX) or sham surgery.

| **Tukey's multiple comparisons test** | **Mean diff,** | **95,00% CI of diff,** | **Summary** | **Adjusted P Value** |
| --- | --- | --- | --- | --- |
|  |  |  |  |  |
| Day -2 |  |  |  |  |
| **SHAM+IgG2a vs. VX+IgG2a** | -0,43 | -1,56 to 0,69 | ns | 0,742 |
| **SHAM+IgG2a vs. SHAM+anti-Ly6G** | -0,10 | -1,22 to 1,02 | ns | 0,996 |
| **SHAM+IgG2a vs. VX+anti-Ly6G** | -0,60 | -1,72 to 0,52 | ns | 0,502 |
| **VX+IgG2a vs. SHAM+anti-Ly6G** | 0,33 | -0,79 to 1,46 | ns | 0,864 |
| **VX+IgG2a vs. VX+anti-Ly6G** | -0,17 | -1,29 to 0,96 | ns | 0,980 |
| **SHAM+anti-Ly6G vs. VX+anti-Ly6G** | -0,50 | -1,62 to 0,62 | ns | 0,648 |
|  |  |  |  |  |
| Day -1 |  |  |  |  |
| **SHAM+IgG2a vs. VX+IgG2a** | -0,10 | -1,22 to 1,02 | ns | 0,996 |
| **SHAM+IgG2a vs. SHAM+anti-Ly6G** | -0,03 | -1,16 to 1,09 | ns | 1,000 |
| **SHAM+IgG2a vs. VX+anti-Ly6G** | 0,10 | -1,02 to 1,22 | ns | 0,996 |
| **VX+IgG2a vs. SHAM+anti-Ly6G** | 0,07 | -1,06 to 1,19 | ns | 0,999 |
| **VX+IgG2a vs. VX+anti-Ly6G** | 0,20 | -0,92 to 1,32 | ns | 0,966 |
| **SHAM+anti-Ly6G vs. VX+anti-Ly6G** | 0,13 | -0,99 to 1,26 | ns | 0,989 |
|  |  |  |  |  |
| Day 0 |  |  |  |  |
| **SHAM+IgG2a vs. VX+IgG2a** | 0,00 | -1,12 to 1,12 | ns | >0,9999 |
| **SHAM+IgG2a vs. SHAM+anti-Ly6G** | 0,00 | -1,12 to 1,12 | ns | >0,9999 |
| **SHAM+IgG2a vs. VX+anti-Ly6G** | 0,00 | -1,12 to 1,12 | ns | >0,9999 |
| **VX+IgG2a vs. SHAM+anti-Ly6G** | 0,00 | -1,12 to 1,12 | ns | >0,9999 |
| **VX+IgG2a vs. VX+anti-Ly6G** | 0,00 | -1,12 to 1,12 | ns | >0,9999 |
| **SHAM+anti-Ly6G vs. VX+anti-Ly6G** | 0,00 | -1,12 to 1,12 | ns | >0,9999 |
|  |  |  |  |  |
| Day 1 |  |  |  |  |
| **SHAM+IgG2a vs. VX+IgG2a** | 1,43 | 0,31 to 2,56 | ** | 0,007 |
| **SHAM+IgG2a vs. SHAM+anti-Ly6G** | 0,37 | -0,76 to 1,49 | ns | 0,827 |
| **SHAM+IgG2a vs. VX+anti-Ly6G** | 3,17 | 2,04 to 4,29 | **** | <0,0001 |
| **VX+IgG2a vs. SHAM+anti-Ly6G** | -1,07 | -2,19 to 0,056 | ns | 0,069 |
| **VX+IgG2a vs. VX+anti-Ly6G** | 1,73 | 0,61 to 2,86 | *** | 0,001 |
| **SHAM+anti-Ly6G vs. VX+anti-Ly6G** | 2,80 | 1,68 to 3,90 | **** | <0,0001 |
|  |  |  |  |  |
| Day 2 |  |  |  |  |
| **SHAM+IgG2a vs. VX+IgG2a** | 2,47 | 1,34 to 3,59 | **** | <0,0001 |
| **SHAM+IgG2a vs. SHAM+anti-Ly6G** | 0,73 | -0,39 to 1,86 | ns | 0,323 |
| **SHAM+IgG2a vs. VX+anti-Ly6G** | 1,70 | 0,57 to 2,82 | *** | 0,001 |
| **VX+IgG2a vs. SHAM+anti-Ly6G** | -1,73 | -2,86 to -0,61 | *** | 0,001 |
| **VX+IgG2a vs. VX+anti-Ly6G** | -0,77 | -1,89 to 0,36 | ns | 0,285 |
| **SHAM+anti-Ly6G vs. VX+anti-Ly6G** | 0,97 | -0,16 to 2,09 | ns | 0,116 |
|  |  |  |  |  |
| Day 3 |  |  |  |  |
| **SHAM+IgG2a vs. VX+IgG2a** | 1,27 | 0,14 to 2,39 | * | 0,021 |
| **SHAM+IgG2a vs. SHAM+anti-Ly6G** | 0,70 | -0,42 to 1,82 | ns | 0,364 |
| **SHAM+IgG2a vs. VX+anti-Ly6G** | 1,27 | 0,14 to 2,39 | * | 0,021 |
| **VX+IgG2a vs. SHAM+anti-Ly6G** | -0,57 | -1,69 to 0,56 | ns | 0,550 |
| **VX+IgG2a vs. VX+anti-Ly6G** | 0,00 | -1,12 to 1,12 | ns | >0,9999 |
| **SHAM+anti-Ly6G vs. VX+anti-Ly6G** | 0,57 | -0,56 to 1,69 | ns | 0,550 |
|  |  |  |  |  |
| Day 4 |  |  |  |  |
| **SHAM+IgG2a vs. VX+IgG2a** | 1,27 | 0,14 to 2,39 | * | 0,021 |
| **SHAM+IgG2a vs. SHAM+anti-Ly6G** | 1,00 | -0,12 to 2,12 | ns | 0,098 |
| **SHAM+IgG2a vs. VX+anti-Ly6G** | 1,17 | 0,04 to 2,29 | * | 0,039 |
| **VX+IgG2a vs. SHAM+anti-Ly6G** | -0,27 | -1,39 to 0,86 | ns | 0,924 |
| **VX+IgG2a vs. VX+anti-Ly6G** | -0,10 | -1,22 to 1,02 | ns | 0,996 |
| **SHAM+anti-Ly6G vs. VX+anti-Ly6G** | 0,17 | -0,96 to 1,29 | ns | 0,980 |
|  |  |  |  |  |
| Day 5 |  |  |  |  |
| **SHAM+IgG2a vs. VX+IgG2a** | 1,03 | -0,09 to 2,16 | ns | 0,082 |
| **SHAM+IgG2a vs. SHAM+anti-Ly6G** | 0,47 | -0,66 to 1,59 | ns | 0,696 |
| **SHAM+IgG2a vs. VX+anti-Ly6G** | 1,10 | -0,023 to 2,22 | ns | 0,057 |
| **VX+IgG2a vs. SHAM+anti-Ly6G** | -0,57 | -1,69 to 0,56 | ns | 0,550 |
| **VX+IgG2a vs. VX+anti-Ly6G** | 0,07 | -1,06 to 1,19 | ns | 0,999 |
| **SHAM+anti-Ly6G vs. VX+anti-Ly6G** | 0,63 | -0,49 to 1,76 | ns | 0,454 |
|  |  |  |  |  |
| Day 6 |  |  |  |  |
| **SHAM+IgG2a vs. VX+IgG2a** | 1,17 | 0,05 to 2,29 | * | 0,039 |
| **SHAM+IgG2a vs. SHAM+anti-Ly6G** | 0,63 | -0,49 to 1,76 | ns | 0,454 |
| **SHAM+IgG2a vs. VX+anti-Ly6G** | 1,27 | 0,14 to 2,39 | * | 0,021 |
| **VX+IgG2a vs. SHAM+anti-Ly6G** | -0,53 | -1,66 to 0,59 | ns | 0,599 |
| **VX+IgG2a vs. VX+anti-Ly6G** | 0,10 | -1,02 to 1,22 | ns | 0,996 |
| **SHAM+anti-Ly6G vs. VX+anti-Ly6G** | 0,63 | -0,49 to 1,76 | ns | 0,454 |
|  |  |  |  |  |
| Day 7 |  |  |  |  |
| **SHAM+IgG2a vs. VX+IgG2a** | 1,40 | 0,28 to 2,52 | ** | 0,008 |
| **SHAM+IgG2a vs. SHAM+anti-Ly6G** | 0,47 | -0,66 to 1,59 | ns | 0,696 |
| **SHAM+IgG2a vs. VX+anti-Ly6G** | 1,17 | 0,05 to 2,29 | * | 0,039 |
| **VX+IgG2a vs. SHAM+anti-Ly6G** | -0,93 | -2,06 to 0,19 | ns | 0,137 |
| **VX+IgG2a vs. VX+anti-Ly6G** | -0,23 | -1,36 to 0,89 | ns | 0,948 |
| **SHAM+anti-Ly6G vs. VX+anti-Ly6G** | 0,70 | -0,42 to 1,82 | ns | 0,364 |

**Table S4.** Tukey's multiple comparisons test weight of mice following sustained extracellular Ly6G ligation (anti-Ly6G), isotype control (IgG2a), and vagotomy (VX) or sham surgery.

| Tukey's multiple comparisons test | Mean diff. | 95.00% CI of diff. | Summary | Adjusted P Value |
| --- | --- | --- | --- | --- |
|  |  |  |  |  |
| Day -2 |  |  |  |  |
| SHAM+IgG2a vs. VX+IgG2a | -0.20 | -0.95 to 0.55 | ns | 0.900 |
| SHAM+IgG2a vs. SHAM+anti-Ly6G | 0.27 | -0.48 to 1.02 | ns | 0.794 |
| SHAM+IgG2a vs. VX+anti-Ly6G | -0.70 | -1.45 to 0.050 | ns | 0.077 |
| VX+IgG2a vs. SHAM+anti-Ly6G | 0.47 | -0.28 to 1.22 | ns | 0.374 |
| VX+IgG2a vs. VX+anti-Ly6G | -0.50 | -1.25 to 0.25 | ns | 0.313 |
| SHAM+anti-Ly6G vs. VX+anti-Ly6G | -0.97 | -1.72 to -0.22 | ** | 0.005 |
|  |  |  |  |  |
| Day -1 |  |  |  |  |
| SHAM+IgG2a vs. VX+IgG2a | 0.30 | -0.45 to 1.05 | ns | 0.728 |
| SHAM+IgG2a vs. SHAM+anti-Ly6G | 0.35 | -0.40 to 1.10 | ns | 0.622 |
| SHAM+IgG2a vs. VX+anti-Ly6G | -0.03 | -0.78 to 0.72 | ns | 0.999 |
| VX+IgG2a vs. SHAM+anti-Ly6G | 0.05 | -0.70 to 0.80 | ns | 0.998 |
| VX+IgG2a vs. VX+anti-Ly6G | -0.33 | -1.08 to 0.42 | ns | 0.658 |
| SHAM+anti-Ly6G vs. VX+anti-Ly6G | -0.38 | -1.13 to 0.37 | ns | 0.549 |
|  |  |  |  |  |
| Day 0 |  |  |  |  |
| SHAM+IgG2a vs. VX+IgG2a | 0.00 | -0.75 to 0.75 | ns | >0.999 |
| SHAM+IgG2a vs. SHAM+anti-Ly6G | 0.00 | 0.75 to 0.75 | ns | >0.999 |
| SHAM+IgG2a vs. VX+anti-Ly6G | 0.00 | 0.75 to 0.75 | ns | >0.999 |
| VX+IgG2a vs. SHAM+anti-Ly6G | 0.00 | 0.75 to 0.75 | ns | >0.999 |
| VX+IgG2a vs. VX+anti-Ly6G | 0.00 | 0.75 to 0.75 | ns | >0.999 |
| SHAM+anti-Ly6G vs. VX+anti-Ly6G | 0.00 | 0.75 to 0.75 | ns | >0.999 |
|  |  |  |  |  |
| Day 1 |  |  |  |  |
| SHAM+IgG2a vs. VX+IgG2a | 0.90 | 0.15 to 1.65 | * | 0.012 |
| SHAM+IgG2a vs. SHAM+anti-Ly6G | 0.13 | -0.62 to 0.88 | ns | 0.967 |
| SHAM+IgG2a vs. VX+anti-Ly6G | 2.58 | 1.83 to 3.33 | **** | <0.0001 |
| VX+IgG2a vs. SHAM+anti-Ly6G | -0.77 | -1.52 to -0.017 | * | 0.0431 |
| VX+IgG2a vs. VX+anti-Ly6G | 1.68 | 0.93 to 2.43 | **** | <0.0001 |
| SHAM+anti-Ly6G vs. VX+anti-Ly6G | 2.45 | 1.70 to 3.20 | **** | <0.0001 |
|  |  |  |  |  |
| Day 2 |  |  |  |  |
| SHAM+IgG2a vs. VX+IgG2a | 1.07 | 0.32 to 1.82 | ** | 0.002 |
| SHAM+IgG2a vs. SHAM+anti-Ly6G | 0.25 | -0.50 to 1.00 | ns | 0.824 |
| SHAM+IgG2a vs. VX+anti-Ly6G | 1.92 | 1.17 to 2.67 | **** | <0.0001 |
| VX+IgG2a vs. SHAM+anti-Ly6G | -0.82 | -1.57 to -0.07 | * | 0.027 |
| VX+IgG2a vs. VX+anti-Ly6G | 0.85 | 0.10 to 1.60 | * | 0.019 |
| SHAM+anti-Ly6G vs. VX+anti-Ly6G | 1.67 | 0.92 to 2.42 | **** | <0.0001 |
|  |  |  |  |  |
| Day 3 |  |  |  |  |
| SHAM+IgG2a vs. VX+IgG2a | 0.88 | 0.13 to 1.63 | * | 0.014 |
| SHAM+IgG2a vs. SHAM+anti-Ly6G | -0.02 | -0.77 to 0.73 | ns | >0.999 |
| SHAM+IgG2a vs. VX+anti-Ly6G | 1.08 | 0.33 to 1.83 | ** | 0.002 |
| VX+IgG2a vs. SHAM+anti-Ly6G | -0.90 | -1.65 to -0.15 | * | 0.012 |
| VX+IgG2a vs. VX+anti-Ly6G | 0.20 | -0.55 to 0.95 | ns | 0.901 |
| SHAM+anti-Ly6G vs. VX+anti-Ly6G | 1.10 | 0.35 to 1.85 | ** | 0.001 |
|  |  |  |  |  |
| Day 4 |  |  |  |  |
| SHAM+IgG2a vs. VX+IgG2a | 0.95 | 0.20 to 1.70 | ** | 0.007 |
| SHAM+IgG2a vs. SHAM+anti-Ly6G | -0.08 | -0.83 to 0.67 | ns | 0.992 |
| SHAM+IgG2a vs. VX+anti-Ly6G | 1.08 | 0.33 to 1.83 | ** | 0.001 |
| VX+IgG2a vs. SHAM+anti-Ly6G | -1.03 | -1.78 to -0.28 | ** | 0.003 |
| VX+IgG2a vs. VX+anti-Ly6G | 0.13 | -0.62 to 0.88 | ns | 0.968 |
| SHAM+anti-Ly6G vs. VX+anti-Ly6G | 1.17 | 0.42 to 1.92 | *** | 0.001 |
|  |  |  |  |  |
| Day 5 |  |  |  |  |
| SHAM+IgG2a vs. VX+IgG2a | 0.88 | 0.13 to 1.63 | * | 0.014 |
| SHAM+IgG2a vs. SHAM+anti-Ly6G | -0.02 | -0.77 to 0.73 | ns | >0.999 |
| SHAM+IgG2a vs. VX+anti-Ly6G | 0.50 | -0.25 to 1.25 | ns | 0.313 |
| VX+IgG2a vs. SHAM+anti-Ly6G | -0.90 | -1.65 to -0.15 | * | 0.012 |
| VX+IgG2a vs. VX+anti-Ly6G | -0.38 | -1.13 to 0.37 | ns | 0.549 |
| SHAM+anti-Ly6G vs. VX+anti-Ly6G | 0.52 | -0.23 to 1.27 | ns | 0.284 |
|  |  |  |  |  |
| Day 6 |  |  |  |  |
| SHAM+IgG2a vs. VX+IgG2a | 1.07 | 0.31 to 1.82 | ** | 0.002 |
| SHAM+IgG2a vs. SHAM+anti-Ly6G | 0.37 | -0.38 to 1.12 | ns | 0.585 |
| SHAM+IgG2a vs. VX+anti-Ly6G | 0.68 | -0.07 to 1.43 | ns | 0.088 |
| VX+IgG2a vs. SHAM+anti-Ly6G | -0.70 | -1.45 to 0.05 | ns | 0.077 |
| VX+IgG2a vs. VX+anti-Ly6G | -0.38 | -1.13 to 0.37 | ns | 0.549 |
| SHAM+anti-Ly6G vs. VX+anti-Ly6G | 0.32 | -0.43 to 1.07 | ns | 0.694 |
|  |  |  |  |  |
| Day 7 |  |  |  |  |
| SHAM+IgG2a vs. VX+IgG2a | 1.23 | 0.48 to 1.98 | *** | 0.0002 |
| SHAM+IgG2a vs. SHAM+anti-Ly6G | 0.15 | -0.60 to 0.90 | ns | 0.954 |
| SHAM+IgG2a vs. VX+anti-Ly6G | 0.15 | -0.60 to 0.90 | ns | 0.955 |
| VX+IgG2a vs. SHAM+anti-Ly6G | -1.08 | -1.83 to -0.33 | ** | 0.001 |
| VX+IgG2a vs. VX+anti-Ly6G | -1.08 | -1.83 to -0.33 | ** | 0.001 |
| SHAM+anti-Ly6G vs. VX+anti-Ly6G | 0.00 | -0.75 to 0.75 | ns | >0.999 |
